# Morphological, Physiological, and Molecular Responses of Sweetly Fragrant *Luculia gratissima* During the Floral Transition Stage Induced by Short-Day Photoperiod

**DOI:** 10.3389/fpls.2021.715683

**Published:** 2021-08-11

**Authors:** Xiongfang Liu, Youming Wan, Jing An, Xiujiao Zhang, Yurong Cao, Zhenghong Li, Xiuxian Liu, Hong Ma

**Affiliations:** ^1^Research Institute of Resources Insects, Chinese Academy of Forestry, Kunming, China; ^2^College of Forestry, Nanjing Forestry University, Nanjing, China

**Keywords:** *Luculia gratissima*, floral transition, photoperiod, flowering pathway, phytohormone, regulatory network

## Abstract

Photoperiod-regulated floral transition is vital to the flowering plant. *Luculia gratissima* “Xiangfei” is a flowering ornamental plant with high development potential economically and is a short-day woody perennial. However, the genetic regulation of short-day-induced floral transition in *L. gratissima* is unclear. To systematically research the responses of *L. gratissima* during this process, dynamic changes in morphology, physiology, and transcript levels were observed and identified in different developmental stages of long-day- and short-day-treated *L. gratissima* plants. We found that floral transition in *L. gratissima* occurred 10 d after short-day induction, but flower bud differentiation did not occur at any stage under long-day conditions. A total of 1,226 differentially expressed genes were identified, of which 146 genes were associated with flowering pathways of sugar, phytohormones, photoperiod, ambient temperature, and aging signals, as well as floral integrator and meristem identity genes. The trehalose-6-phosphate signal positively modulated floral transition by interacting with SQUAMOSA PROMOTER-BINDING-LIKE PROTEIN 4 (SPL4) in the aging pathway. Endogenous gibberellin, abscisic acid, cytokinin, and jasmonic acid promoted floral transition, whereas strigolactone inhibited it. In the photoperiod pathway, FD, CONSTANS-LIKE 12, and nuclear factors Y positively controlled floral transition, whereas PSEUDO-RESPONSE REGULATOR 7, FLAVIN-BINDING KELCH REPEAT F-BOX PROTEIN 1, and LUX negatively regulated it. SPL4 and pEARLI1 positively affected floral transition. Suppressor of Overexpression of Constans 1 and AGAMOUSLIKE24 integrated multiple flowering signals to modulate the expression of *FRUITFULL*/*AGL8*, *AP1*, *LEAFY*, *SEPALLATAs*, *SHORT VEGETATIVE PHASE*, and *TERMINAL FLOWER 1*, thereby regulating floral transition. Finally, we propose a regulatory network model for short-day-induced floral transition in *L. gratissima*. This study improves our understanding of flowering time regulation in *L. gratissima* and provides knowledge for its production and commercialization.

## Introduction

Floral transition (the switch from vegetative to reproductive development) is a critical stage in the life history of flowering plants, particularly in horticultural ornamental plants (Cho et al., 2017; Shang et al., 2020). This process is regulated by both environmental and endogenous signals (Cho et al., 2017). Recently, major breakthroughs have been made in research on the molecular regulatory networks of floral transition in *Arabidopsis thaliana* (Cruciferae), an annual long-day (LD) photoperiod responsive plant (Liu et al., 2020; Zhang et al., 2020; Lv et al., 2021). In *A. thaliana*, different endogenous (autonomous, gibberellin, circadian rhythm, age, and sugar signals) and environmental (vernalization, temperature, and photoperiod) signals congregate on some floral integrators, such as *SUPPRESSOR OF OVEREXPRESSION OF CONSTANS 1* (*SOC1*), *FLOWERING LOCUS T* (*FT*), and *AGAMOUSLIKE24* (*AGL24*), further activating floral meristem identity genes, such as *LEAFY* (*LFY*) and *APETALA1* (*AP1*), which irreversibly convert vegetative meristem to floral meristem (Blümel et al., 2015). However, there is still much to learn regarding the regulation of floral transition in perennial woody plants. Perennial woody plants do not die after flowering. Instead, they produce new flower buds and vegetative branches annually and have characteristics of long reproductive cycles and seasonal flowering (Khan et al., 2014). Therefore, studies on annual plants cannot completely reveal the floral transition mechanisms in perennial woody plants. There are significant differences in the molecular mechanisms of floral transition in perennial woody plants compared with those of *A. thaliana*. For example, gibberellin (GA) promotes the transition from vegetative to reproductive development in *A. thaliana* but has inhibitory effects in some perennial woody plants (Yamaguchi et al., 2014; Li et al., 2018; Bao et al., 2020). Furthermore, in the study on floral transition mechanisms regulated by light intensity, in contrast to Arabidopsis, which is affected by retrograde signaling from in response to photosynthesis (Feng et al., 2016), cultivated roses are specifically controlled by some light-sensitive transcription factor complexes (Balcerowicz, 2021; Sun et al., 2021). Therefore, it is crucial to accelerate the pace of research on floral transition in perennial woody plants, which is expected to improve our understanding of the differences in floral transition mechanisms in flowering plants with different life histories.

**Figure 1:**
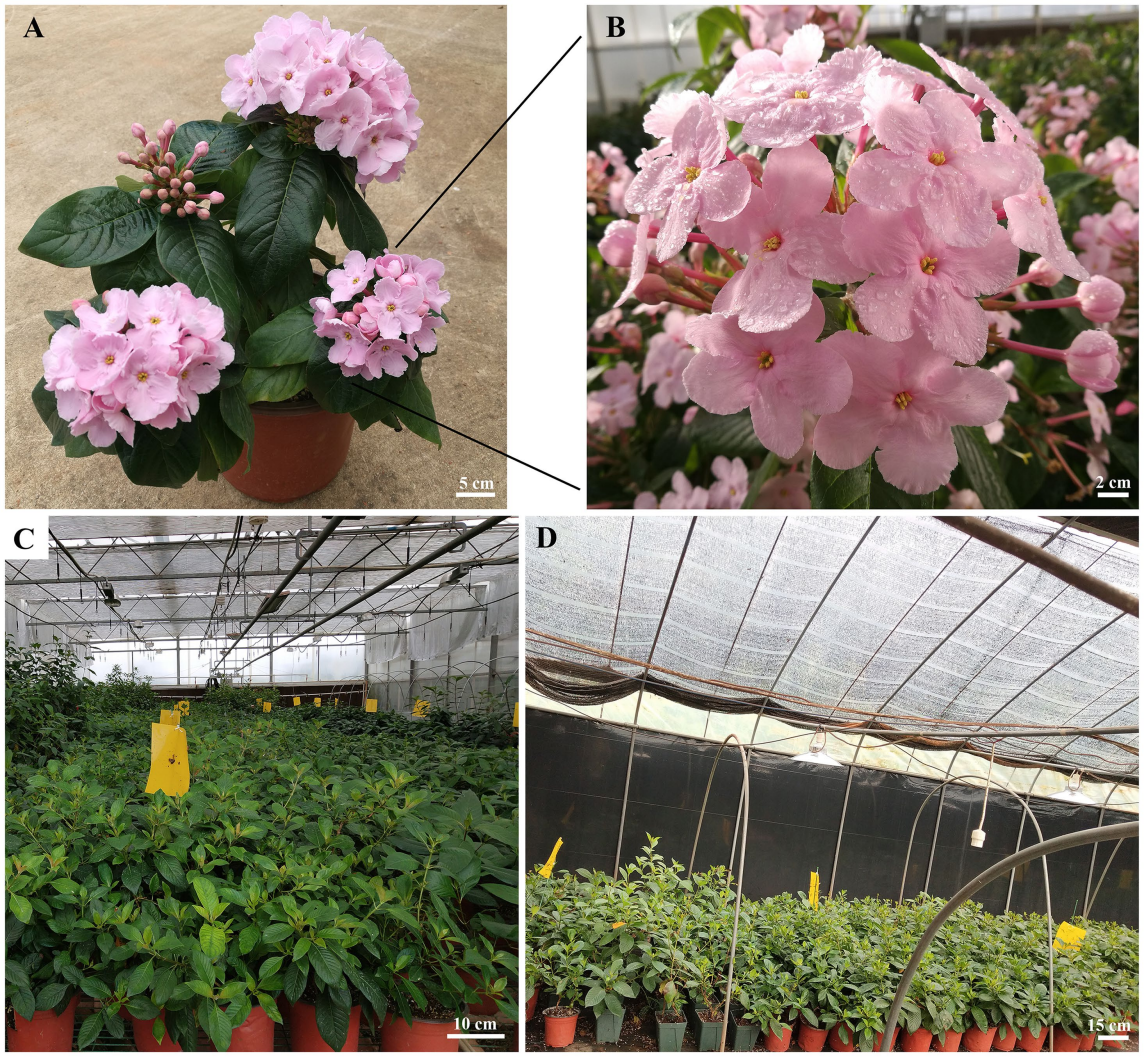
Features of *Luculia gratissima* “Xiangfei” and the overview of greenhouses under two different photoperiods. **(A)** Whole plant of *L. gratissima* “Xiangfei.” **(B)** Flowers of *L. gratissima* “Xiangfei.” **(C)** Greenhouse under night-break treatment. **(D)** Greenhouse under short-day photoperiod.

*Luculia gratissima* (Wall.) Sweet (Rubiaceae) is a perennial evergreen shrub or small tree that is distributed in the southeastern edge of the Tibetan plateau in southwest China and neighboring Nepal and Myanmar (Zhou et al., 2011). *L. gratissima* “Xiangfei,” a new cultivar cultivated by our research team for many years, has pink flowers, a strong fragrance, and a large and dense inflorescence (Figures 1A,B); it is a woody horticultural flower with great ornamental value and economic development potential. In natural conditions, seed-derived plants of the cultivar “Xiangfei” grow for 2 years before flowering, with flowering from August to December every year. However, this plant has not entered the large-scale commercial production stage because of imperfect flowering time regulation techniques. Previous studies showed that the cultivar “Xiangfei” can only complete floral transition at short-day (SD) photoperiods (Wan et al., 2018), and thus, controlling day length to induce flowering is required to achieve year-round production. The species of interest, *L. gratissima*, is in a different clade than that of *A. thaliana*. Thus, mechanistic differences are likely to exist. Therefore, understanding the mechanisms of short-day-induced floral transition in *L. gratissima* “Xiangfei” has important significance for understanding and solving flowering-related problems.

In the present study, we investigated responses of *L. gratissima* during short-day-induced floral transition stage at the morphological, physiological, and transcriptome levels. The aims of this study were as follows: (1) to observe shoot apexes of *L. gratissima* of short-day treatment during five developmental stages using morphological and histological methods to identify the time point of floral transition in *L. gratissima*; (2) to measure endogenous substance contents to study the soluble sugar and hormone effects in floral transition in *L. gratissima*; and (3) to conduct an RNA sequencing (RNA-seq) analysis of the transcriptomes of *L. gratissima* shoot apexes and leaves at four different stages, 7, 10, 13, and 19 days after the initiation of long-day (LD) and short-day (SD) treatments, to study the molecular regulatory mechanism of short-day-induced floral transition in *L. gratissima*. The results presented in this research will aid in regulating *L. gratissima* flowering and achieving year-round production. Additionally, identification of important regulatory genes will provide important guidance for flowering-related molecular breeding in the future.

## Materials and Methods

### Plant Materials, Growth Conditions, and Light Treatments

*Luculia gratissima* cultivar “Xiangfei” cuttings from three-year-old plants were obtained from the central Yunnan Plateau experimental station of Research Institute of Resources Insects, Chinese Academy of Forestry (Yunnan, China; 25°13'N, 102°12'E, 1826 m a.s.l.). In mid-December 2016, cuttings with two stem nodes and shoot apexes were planted in a mixed matrix (peat and perlite at a 3:1 ratio) and grown in an 18–25°C greenhouse under natural lighting. Cuttings with roots were transplanted into pots and maintained in the same greenhouse under natural lighting. To prevent these plants from being induced by SD photoperiod, shoot apical meristems (SAMs) were removed from all plants when 2–3 new stem nodes were formed, and high-pressure sodium lamps were used for additional lighting during 22:00–02:00 (night-break treatment; Figure 1C). In addition, considering the effects of individual developmental age on flowering time (Evans et al., 1992), some plants were placed in the natural environment as controls and the time when flower bud differentiation occurred in these plants was used as the start time for photoperiod treatments. On 10 August 2017 (when flower buds began to appear in some natural control plants), plants with the same number of branches longer than 5 cm were selected from among the night-break treatment plants and then were subjected to either LD (night-break treatment as described above) or SD (10 h light/14 h dark; Figure 1D) for a further 90 days. The light source was supplied using high-pressure sodium lamps. The greenhouse temperature was 20 ± 2°C with approximately 60% relative humidity. Shoot apexes and their surrounding leaves of the main branches of SD and LD plants were sampled during 09:00–11:30 every 3–5 d after the initiation of the photoperiod treatments. For each stage, 10–20 shoot apexes and their surrounding leaves were packed together into each of the 10 biological replicates, of which one biological replicate was rapidly immersed into FAA fixative (50% ethanol: acetic acid: formaldehyde, 18:1:1) for morphological analysis, whereas the remaining nine biological replicates were snap-frozen in liquid nitrogen and then stored at −80°C for measurements of soluble sugar and endogenous hormone contents, as well as RNA extraction.

### Morphological Anatomical Observations

Ten FAA-fixed shoot apexes of SD and LD plants at each stage were made into sections with a thickness of 8–10 μm using paraffin section method (Fischer et al., 2008), and were stained with safranin O-fast green, and then were mounted with neutral resin. Finally, the process of bud development was observed under a Carl Zeiss Axio Scope A1 Microscope (Carl Zeiss Microscopy GmbH, Göttingen, Germany).

### Measurements of Soluble Sugar and Endogenous Hormone Contents

**Figure 2:**
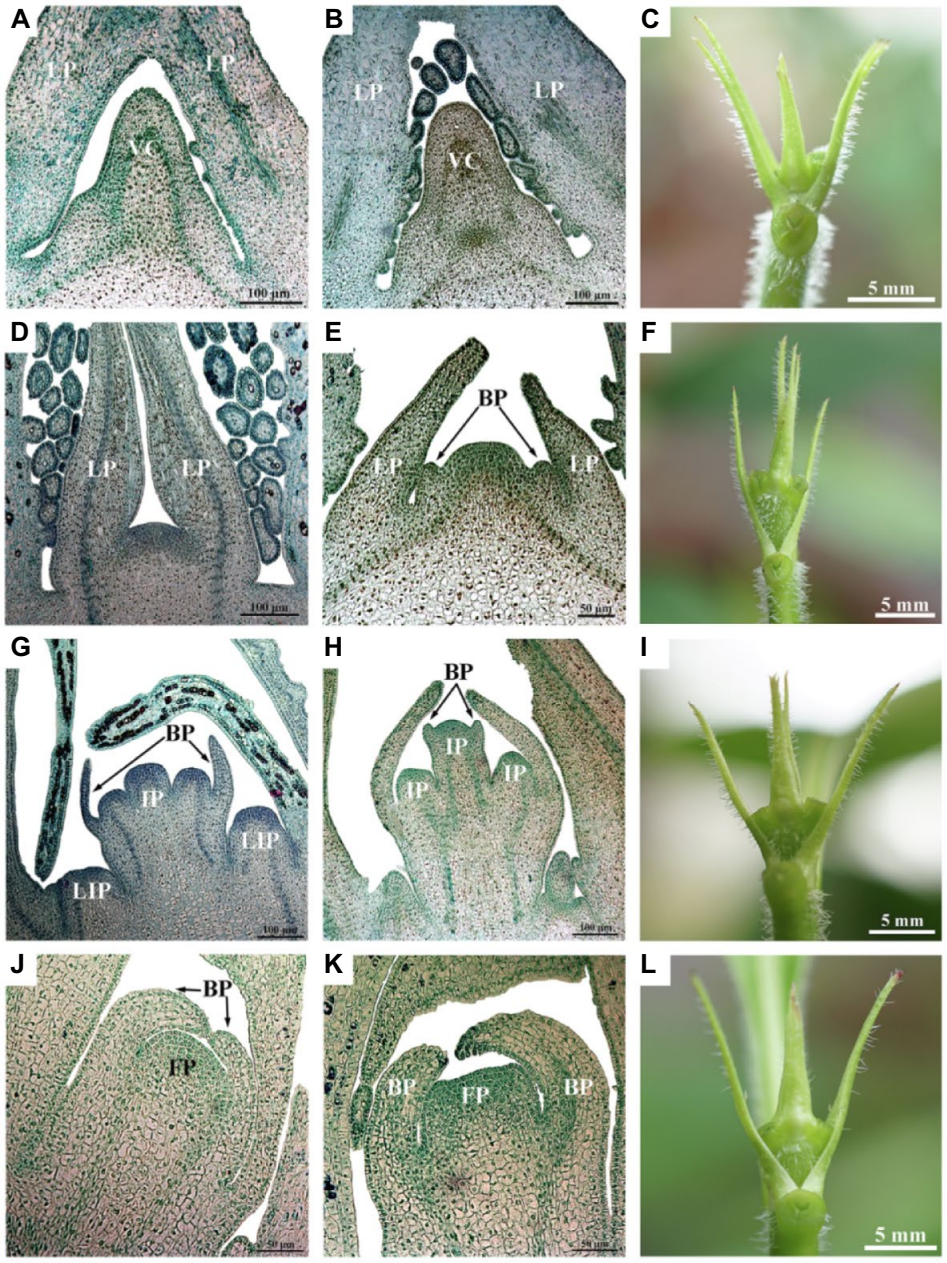
*Luculia gratissima* morphological and histological characteristics, shoot apexes at five time points upon short-day treatment. **(A–C)** Vegetative buds in the undifferentiated stage (SD0 to SD7). **(D–F)** Bract primordial differentiation stage (SD10). **(G–I)** Inflorescence primordial differentiation stage (SD13). **(J–L)** Floret primordial differentiation stage (SD19). **(A,B,D,G,H)** Histological images obtained from paraffin-embedded sectioned samples (scale bar: 100 μm). **(E,J,K)** Histological images obtained from paraffin-embedded sectioned samples (scale bar: 50 μm). **(C,F,I,L)** The external morphology of shoot apexes at different developmental stages (scale bar: 5 mm). BP, bract primordia; FP, floret primordia; IP, inflorescence primordia; LIP, lateral inflorescence primordium; LP, leaf primordia; and VC, vegetative cone.

According to the anatomical observation results, samples from the SD treatment at five stages [0 d (SD0), 7 d (SD7), 10 d (SD10), 13 d (SD13), and 19 d (SD19)] close to flower bud differentiation (Figure 2) were selected for measurements of soluble sugar and endogenous hormone contents of three biological replicates. For each of the three biological replicates from each stage, soluble sugar contents were measured using sulfuric acid-anthrone colorimetric assays as previously reported (Wang et al., 2015), and endogenous hormones [GA_3_, indoleacetic acid (IAA), ABA, and zeatin (ZT)] were quantified with high-performance liquid chromatography-mass spectrometry (Aglient1290, Nanjing, China; AB 6500, Nanjing, China) as previously reported (Pan et al., 2010). Before comparing changes in the soluble sugar and hormone contents among the five stages, the Shapiro–Wilk test and Levene test were used to analyze the normality and homogeneity of variance of each dataset. Because the four sets of data did not follow a normal distribution (*p* < 0.05), a Kruskal-Wallis *H* test was employed for analysis of significant differences, and false discovery rate (Benjamini and Yekutieli, 2001) was used for the multiple testing correction of significant *p*-values. Additionally, the Tukey–Kramer method was used for post-hoc testing of soluble sugar and hormone contents at the five stages. The above analyses were performed in the “car” and “stats” packages in R software and the data were expressed as the mean ± SD.

### Transcriptome Sequencing and Data Analysis

Likewise, based on the anatomical observation results, samples from the SD and LD treatments at the four stages [7 d (SD7 or LD7), 10 d (SD10 or LD10), 13 d (SD13 or LD13), and 19 d (SD19 or LD19)] close to flower bud differentiation of SD plants (Figure 2) were selected for RNA extraction. Total RNA extracted from each of the three biological replicates was divided into two parts, of which one was used for RNA-seq and the other was used for quantitative real-time PCR (qRT-PCR) validation. Total RNA was extracted with the plant total RNA Kit (Tiangen, Beijing, China) following manufacturer’s instructions. The cDNA library construction and paired-end sequencing were conducted with an Illumina HiSeq^™^ 4,000 (Illumina, San Diego, California, United States) at the Gene Denovo Biotechnology Company (Guangzhou, China). The generated raw reads were filtered by removing adapter sequences and ambiguous reads (*N* > 10%) and low-quality reads (more than 40% of bases with value of *Q* ≤ 20) to obtain high-quality clean reads. Without reference genome, clean reads were *de novo* assembled as a transcriptome reference database for *L. gratissima* via Trinity software (Grabherr et al., 2011). Furthermore, clean reads were mapped to ribosome RNA (rRNA) to identify residual rRNA reads. The rRNA removed reads were further mapped to the reference transcriptome using short reads alignment tool Bowtie2 (Langmead et al., 2009) by default parameters. The reference transcriptome unigenes without rRNA reads were generated for next analysis.

All non-redundant unigenes were aligned with selected cutoffs of value of *E* ≤ 1e-05 to six protein databases, including the NR (the NCBI non-redundant protein databases), KOG (EuKaryotic Orthologous Groups), Kyoto Encyclopedia of Genes and Genomes, Swiss-Prot, evolutionary genealogy of genes: Non-supervised Orthologous Groups, and Protein families database of alignments and hidden Markov models. Based on the NR annotation results, these unigenes were also annotated for GO (Gene Ontology) using the Blast2GO software (Conesa et al., 2005), and then GO functional classification of unigenes was obtained by the WEGO software (Ye et al., 2006).

### qRT-PCR Analysis

qRT-PCR was conducted on nine flowering-related unigenes in this study, including *COP1* (Unigene0031506), *ZTL* (Unigene0041339), *FKF1* (Unigene0038380), *GI* (Unigene0051409), *ELF3* (Unigene0051761), *PRR1* (Unigene0045946), *PRR7* (Unigene0003564), *PRR5* (Unigene0047475), and *LHY* (Unigene0035686). To accurately measure gene expression levels, the *ACT7/EF1-α* combination obtained from the past screening was used as an internal reference gene for standardization and correction (Supplementary Data). Primer3 software (Rozen and Skaletsky, 2000) was used to design specific primers for each gene (Supplementary Table S1). The KR106 FastQuantity RT Kit (with gDNase; Tiangen, Beijing, China) was used for reverse transcription of 1 μg total RNA into cDNA according to the manufacturer’s instructions. The StepOnePlus^™^ Real-Time PCR System (Thermo Scientific, Wilmington, DE, United States) was used for qRT-PCR in a 20 μl reaction system, including 4 μl of 50 ng cDNA template, 10 μl of 2 × qPCR Master Mix (Tiangen, Beijing, China), 0.4 μl each of 10 μm forward and reverse primers, and 5.2 μl ddH_2_O. The qRT-PCR amplification conditions were as follows: pre-denaturation at 95°C for 90 s, followed by 40 cycles of denaturation at 95°C for 5 s, annealing at 60°C for 15 s, and extension at 72°C for 20 s, followed by a final extension step at 72°C for 5 min, after amplification, a 65–95°C melting curve analysis was conducted to measure product specificity. The 2^−ΔΔCt^ method (Livak and Schmittgen, 2001) was used to calculate the relative expression levels of the genes in the qRT-PCR experiment. The normalization of gene expression was conducted using the geometric mean of two internal reference genes, *ACT7* and *EF1-α* (Vandesompele et al., 2002).

### Identification and Functional Enrichment of DEGs

The Reads Per kb per Million reads (RPKM) method was used to evaluate unigene expression levels (Mortazavi et al., 2008). Pairwise comparisons were conducted between LD and SD samples to identify differentially expressed genes (DEGs) in response to SD photoperiod during the floral transition process in *L. gratissima*. To generate accurate log_2_foldchange estimates, EdgeR package version 3.8 (Robinson et al., 2010) was used. The thresholds for differential expression were set at fold change 2 (log_2_foldchange = 1) and FDR value cutoff 0.05.

The Mercator online tool^1^ was employed for gene function predictions for the DEGs with a BLAST-CUTOFF of 50. The obtained mapping files were uploaded to MapMan version 3.6 (Thimm et al., 2004) for the functional analysis of DEGs. Wilcoxon rank-sum test was used to analyze the log_2_foldchange of DEGs in each comparison before MapMan version 3.6 (Thimm et al., 2004) was used for visualization of the results.

### Co-expression Network Analysis

Weighted gene co-expression network analysis (WGCNA; Langfelder and Horvath, 2008) was employed to generate the co-expression network modules of DEGs. The parameter settings used were soft threshold = 20, minModuleSize = 30, TOMType = signed, and mergeCutHeight = 0.25, and default values were used for the remaining parameters. The eigengene value of every module was calculated and the associations between every gene in eight samples were tested. KOBAS 3.0 (Xie et al., 2011) was used for GO enrichment analysis of genes in the clustering modules. Cytoscape version 3.7.1 (Shannon et al., 2003) was used for visualization of the co-expression network.

## Results

### Morphological Differentiation of Shoot Apexes During Floral Transition

*Luculia gratissima* cultivar “Xiangfei” cuttings from three-year-old plants were planted and grown for about 8 months before photoperiod treatments. When some flower buds appeared in natural control plants, the generated cutting plants were transferred to SD conditions (10 h light/14 h dark, 20 ± 2°C, 60% relative humidity) or LD conditions (night-break treatment for 4 h, 20 ± 2°C, 60% relative humidity). Shoot apexes and their surrounding leaves of the main branches of SD and LD plants were sampled during 09:00–11:30 every 3–5 d after the initiation of the photoperiod treatments.

The morphological differentiation of *L. gratissima* shoot apexes was observed through paraffin sections. The results showed that 0 d to 7 d under the SD treatment (SD0 to SD7) was the vegetative growth stage (undifferentiated stage), in which the tip of the growth cone in the bud was narrow and pointed and surrounded by leaf primordia (Figures 2A–C). At 10 d after the initiation of the SD treatment (SD10), the bract primordial differentiation stage began (Figures 2D–F). In this stage, the growth cone of the bud appeared dome shape; subsequently, the dome-shaped growth cone began broadening and flattening, and the bract primordia along the periphery were formed, which was an important marker of the transition from vegetative growth to reproductive growth (Figures 2D–F). At 13 d after the initiation of the SD treatment (SD13), the inflorescence primordial differentiation stage began. At this stage, the growth cone in the bract primordia elongated to form three hemispherical protrusions, i.e., inflorescence primordia. Simultaneously, the lateral base of the bract primordia differentiated into lateral inflorescence primordia. Next, bilateral protrusions at each hemispherical inflorescence primordium differentiated into bract inflorescences (Figures 2G–I). At 19 d after the initiation of the SD treatment (SD19), the floret primordial differentiation stage began and a single inflorescence primordium in the bract primordia gradually widened to become floret primordia at the tip of the bud (Figures 2J–L). These results showed that the floral transition period began 10 d after the initiation of the SD treatment, and the selection of time points before and after this period could facilitate the physiological study of floral transition. However, the buds of LD plants were at vegetative growth stage all the time (Supplementary Figure S1). Therefore, the LD treatment was used as a control in this study, and 7 d (SD7), 10 d (SD10), 13 d (SD13), and 19 d (SD19) in the SD treatment were selected to study the physiological and molecular regulation patterns of floral transition. LD samples (i.e., LD7, LD10, LD13, and LD19) for RNA-seq analysis were taken in parallel at the same time points as respective SD samples.

### Dynamic Changes in Endogenous Substance Content During Floral Transition

**Figure 3:**
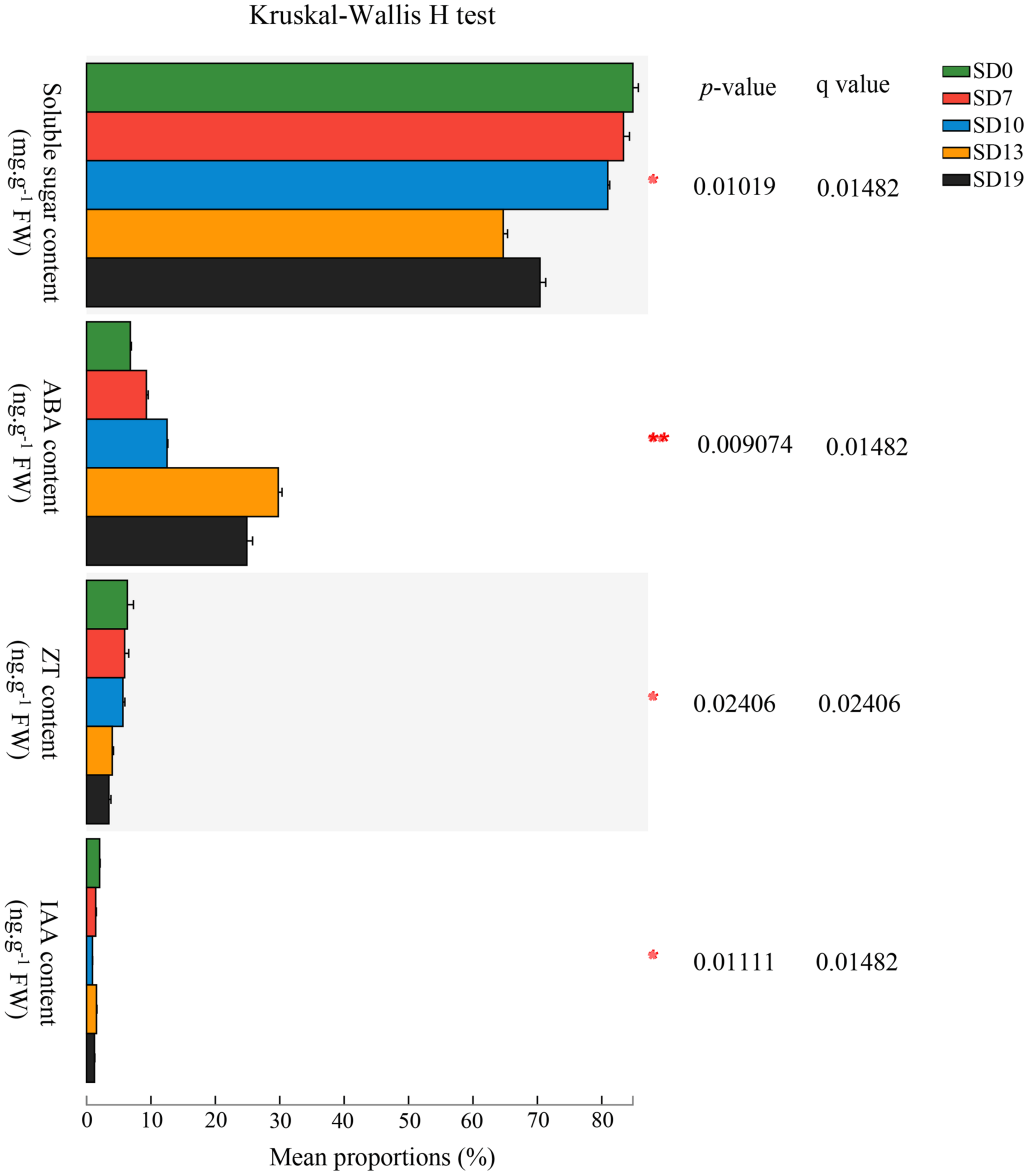
*Luculia gratissima* endogenous soluble sugar content and hormonal changes, shoot apexes and leaves at five stages upon short-day treatment. The y-axis shows soluble sugar and four hormones, and the x-axis shows the average relative abundance of the endogenous soluble sugars and hormones. Colored columns represent different developmental stages. ^*^0.01 < *p* ≤ 0.05; ^**^0.001 < *p* ≤ 0.01.

Contents of soluble sugars and endogenous hormones [gibberellin (GA_3_), IAA, abscisic acid (ABA), and zeatin (ZT)] were measured at 0 d (SD0), 7 d (SD7), 10 d (SD10), 13 d (SD13), and 19 d (SD19) after the initiation of the SD treatment. The Kruskal-Wallis *H* test results showed that except for GA_3_, which could not be detected because it was below the limit of quantitation (0.1 ng/ml), there were significant differences in the contents of the other substances among the five stages (adjusted *p* < 0.05; Figure 3). Soluble sugar, ZT, and IAA reached their peaks at SD0, which were 28.86 ± 0.67 mg g^−1^ FW, 2.15 ± 0.30 ng g^−1^ FW, and 0.69 ± 0.04 ng g^−1^ FW, respectively. Additionally, soluble sugar and ZT decreased from SD0 to SD19, indicating that the soluble sugar and ZT contents in SAMs of *L. gratissima* were maintained at a relatively low level during the flowering process. Interestingly, IAA showed an increase in SD13 before decreasing. Similarly, ABA initially increased from SD0 to SD13 and subsequently declined.

Additionally, post-hoc results showed that there were extremely significant differences in the pairwise comparisons between the five time points for ABA (*p* < 0.001). IAA only showed no significant differences between SD7 and SD13 (*p* > 0.1). Soluble sugar did not show any significant differences between SD0 and SD7 (*p* > 0.1). ZT did not show any significant differences between SD0 and SD10, between SD7 and SD10, or between SD13 and SD19 (*p* > 0.1). From these results, it can be seen that ABA levels changed rapidly and dynamically over these five stages, whereas ZT levels exhibited little change over the same period. Changes in soluble sugar content mainly occurred in later periods (SD13 to SD19). In contrast to these substances, IAA changes were relatively constant (Figure 3).

### RNA-Seq and qRT-PCR Identification of DEGs

Transcriptomes were generated for three biological replicates from the SD and LD treatments at each of the time points corresponding to the four stages of bud differentiation in SD treatment plants, i.e., at 7 d, 10 d, 13 d, and 19 d (Figure 2), yielding a total of 24 transcriptomes. A total of 1,236,426,670 raw sequencing reads were generated from 24 samples, 1.2 × 10^9^ high-quality clean reads (181Gb) were obtained after filtering, with mean Q20, Q30, and GC contents of 99.11, 97.18, and 43.53%, respectively (Supplementary Table S2). A total of 79,870 unigenes (≥ 200 b) were generated from *de novo* assembly, and the N50 length was 2,118 bp (Supplementary Table S3). Among these unigenes, 35,725 unigenes (44.73%) were successfully annotated to at least one database (Supplementary Figure S2).

With RNA from the same 24 samples used for transcriptome generation, qRT-PCR was conducted for nine flowering-related unigenes identified in through RNA-seq, including *COP1* (Unigene0031506), *ZTL* (Unigene0041339), *FKF1* (Unigene0038380), *GI* (Unigene0051409), *ELF3* (Unigene0051761), *PRR1* (Unigene0045946), *PRR7* (Unigene0003564), *PRR5* (Unigene0047475), and *LHY* (Unigene0035686). The results of qRT-PCR showed that except for *PRR5* (Unigene0047475), the expression patterns of the other eight genes were generally consistent with the RNA-seq data (Supplementary Figure S3), indicating that the transcriptome data generated in this study were reliable and valid. The inconsistency between the relative expression and RPKM values of *PRR5* occurred in LD7 and SD7 samples, and for the possible reasons of this inconsistency, on the one hand, it could be that *PRR5* was not a DEG in the RNA-seq data, and on the other hand, the RPKM values of *PRR5* were lower than 10 in both LD7 and SD7 samples, in which there could be false positives.

A total of 113 (SD7-vs.-LD7), 420 (SD10-vs.-LD10), 483 (SD13-vs.-LD13), and 464 (SD19-vs.-LD19) DEGs were obtained by comparing the LD and SD treatments (Supplementary Figure S4 and Table S4). A total of 1,226 DEGs were identified from these four comparisons, of which five DEGs were shared by four comparisons, and 250 DEGs were present in more than one comparison. There were 110, 302, 288, and 276 stage-specific DEGs in SD7-vs.-LD7, SD10-vs.-LD10, SD13-vs.-LD13, and SD19-vs.-LD19, respectively (Supplementary Figure S4).

### Functional Classifications of DEGs

MapMan is an effective tool for systematic analysis of plant transcriptome metabolic pathways and other biological processes (Ramšak et al., 2013). We employed MapMan to overview transcriptional changes in regulatory, metabolic, and cellular response-related genes (Supplementary Table S5). In “regulation overview,” more DEGs were detected in the other three comparisons contrasted with SD7-vs.-LD7, showing that the physiological and molecular characteristics after flower bud differentiation (SD10, SD13, and SD19) were significantly different from that before flower bud differentiation (SD7). In the IAA metabolic subclass, more DEGs were upregulated in SD19-vs.-LD19 compared with SD7-vs.-LD7, SD10-vs.-LD10, and SD13-vs.-LD13 (Supplementary Figure S5). Yet, IAA content increased from SD10 to SD13 to continue decreasing afterward. Anyhow, the differences between dates were small, although significant (Figure 3). Therefore, IAA was not a key factor mediating floral transition in *L. gratissima*. ABA metabolism-related DEGs were significantly upregulated in all four comparisons (Supplementary Figure S5), and ABA levels were overall increasing in the process of floral transition (Figure 3), demonstrating that ABA could promote floral transition in *L. gratissima*. In “minor CHO metabolism”, trehalose biosynthesis-related DEGs were only upregulated in SD7-vs.-LD7 (Supplementary Figure S6). “Cellular response overview” showed that more development-related DEGs were upregulated in SD10-vs.-LD10 compared with the other three comparisons (Supplementary Figure S7), indicating that these DEGs promoted floral transition in *L. gratissima*.

### Co-expression Module Analysis for DEGs

**Figure 4:**
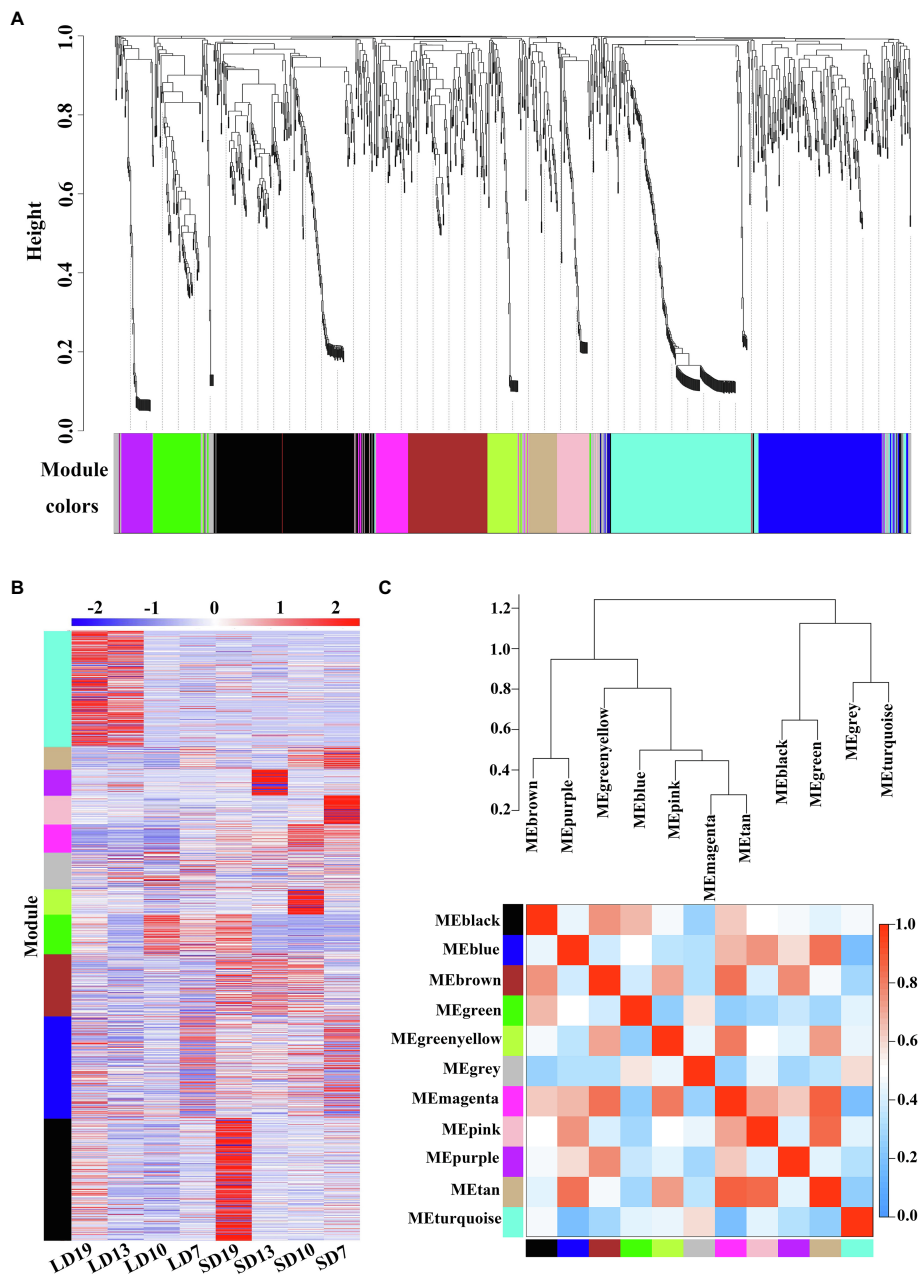
Weighted co-expression network analysis of 1,226 DEGs at four developmental stages of *L. gratissima*, short- or long-day treatments. **(A)** Hierarchical cluster tree showing the co-expression modules, with each tree leaf representing one gene. The major tree branches constitute 11 modules labeled by different colors. **(B)** Heat map of gene relative expression of different modules (y-axis) in eight samples (x-axis). The Z-score normalized RPKM value for an individual gene at a given developmental stage is indicated in a green (low expression) to red (high expression) scale. **(C)** Eigengene network representing the relationships among the different modules. The hierarchical clustering dendrogram of the eigengenes shows the relationships among the modules, whereas the heat map shows the correlation between the different modules, with deeper red color representing a stronger correlation.

WGCNA is a systems biology method for analyzing the correlation relationships between genes in multiple samples (Langfelder and Horvath, 2008). In this study, the results of WGCNA showed that 1,226 DEGs in eight samples were clustered in 11 different co-expression modules (labeled with different colors; Figure 4A). It is noteworthy that four out of 11 co-expression modules significantly correlated with a single sample (*r* > 0.9, *p* < 0.05; Figure 4B and Supplementary Table S6). For example, the largest module (black module) included 247 (20.15%) SD19-specific DEGs (Figure 4B and Supplementary Table S6A).

We further conducted GO enrichment analysis on 11 co-expression modules, and only the greenyellow module was not significantly enriched for any GO terms (Supplementary Table S7). Some GO terms were specifically identified in only a single module. For example, 120 specific GO terms were identified in the black module, which mainly involved signal transduction and negative regulation of metabolic processes, and 34 module-specific GO terms were identified in the brown module, which was mainly associated with growth and development (Supplementary Table S7). However, several GO terms, including “response to organic substance” and “response to a stimulus,” appeared in multiple modules (Supplementary Table S7), indicating possible module-gene interactions. Overall, the extensively enriched GO terms showed that multiple biological processes were involved in the floral transition in *L. gratissima*.

The 11 modules were divided into seven categories based on the correlations between modules (Figure 4C). The heat map showed that there was a high correlation between the blue, magenta, pink, and tan modules, in which the genes were highly expressed in SD7 and SD10 (Figures 4B,C), and were significantly enriched in multiple GO terms involving secondary metabolite biosynthesis, signal transduction, and regulation of developmental processes (Supplementary Table S7).

### Identification of DEG Expression Patterns Associated With Floral Transition in *L. gratissima*

According to the above functional classifications and WGCNA of these DEGs, and flowering-related genes previously reported in model plants (such as *A. thaliana*; Blümel et al., 2015; Bao et al., 2020), a total of 146 unigenes were identified as homologous genes related to floral transition in *L. gratissima*, involving several flowering pathways: sugar metabolism, hormone metabolism and signal transduction, photoperiod, ambient temperature, aging pathways, floral integrator, and floral meristem identity genes. Among these floral transition-related homologous genes, stage-specific DEGs, and common DEGs in SD7-vs.-LD7, SD10-vs.-LD10, SD13-vs.-LD13, and SD19-vs.-LD19, are listed in Supplementary Table S8.

### The Expression Pattern of Sugar Signal-Related Homologs

**Figure 5:**
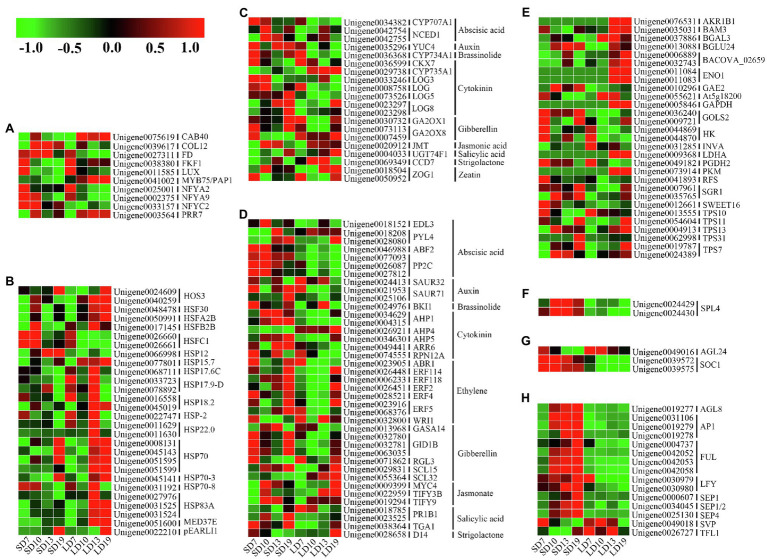
Expression profiles of genes associated with *L. gratissima* floral transition at four developmental stages, short- or long-day treatments. Relative expression profile of **(A)** photoperiod pathway-related genes, **(B)** ambient temperature pathway-related genes, **(C)** phytohormone metabolism-related genes, **(D)** phytohormone signal transduction-related genes, **(E)** sugar signal-related genes, **(F)** aging pathway-related genes, **(G)** floral integrator-related genes, and **(H)** floral meristem identity genes. The Z-score normalized RPKM value for an individual gene at a given developmental stage is represented in a green (low expression) to red (high expression) scale.

The sugar signal pathway, which responds to the sugar budget in plants, is one of the important pathways mediating the transition from vegetative to floral meristems (Blümel et al., 2015). A total of 29 (19.86%) DEGs associated with sugar signal-related genes were identified, involving 23 sugar signal-related homologs. These genes expressed differently in different development stages of *L. gratissima*. For example, *HEXOKINASE* (*HK*) homologs (Unigene0044869 and Unigene0044870) were significantly upregulated in SD7-vs.-LD7 and SD13-vs.-LD13, and a *BETA-GLUCOSIDASE 24* homolog (Unigene0013088) was significantly upregulated in SD10-vs.-LD10. Meanwhile, Unigene0009721 and Unigene0041893, homologs of *GALACTINOL SYNTHASE 2* and *RAFFINOSE SYNTHASE* participating in raffinose synthesis, were upregulated in SD7-vs.-LD7. In addition, *TREHALOSE-6-PHOSPHATE SYNTHSE* (*TPS*) homologs (Unigene0019787, Unigene0024389, Unigene0013555, Unigene0054604, Unigene0004913, and Unigene0062998) were upregulated at various stages, and *SWEET16* homolog (Unigene0012661) was significantly upregulated in SD7-vs.-LD7 and SD10-vs.-LD10 (Figure 5E and Supplementary Table S9). Hence, these genes may directly or indirectly participate in floral transition in *L. gratissima*.

### The Expression Patterns of Phytohormone Metabolism and Signal Transduction Homologs

Many studies have demonstrated that various phytohormones participate in the regulation of floral transition (Shu et al., 2018; Lin et al., 2019; Zhang et al., 2019; Bao et al., 2020). A total of 20 (13.70%) DEGs associated with phytohormone metabolism were identified, and these involved 16 phytohormone metabolism homologous genes and were related to nine phytohormone metabolism pathways. Among these genes, *GIBBERELLIN 2-BETA-DIOXYGENASE 1* (*GA2OX1*) homologs (Unigene0030732) and *GA2OX8* homologs (Unigene0073113), which are involved in GA metabolism, were significantly upregulated in SD10-vs.-LD10 and/or SD19-vs.-LD19. Meanwhile, Unigene0034382 (*CYP707A1* homolog) and Unigene0042754 and Unigene0042755 (*NCED1* homologs), respectively, encoding abscisic acid (ABA) 8'-hydroxylase 1 and nine-cis-epoxycarotenoid dioxygenase, were significantly upregulated in SD10-vs.-LD10. In addition, a homolog (Unigene0035296) of *YUC4*, encoding indole-3-pyruvate monooxygenase, which mediates auxin biosynthesis, was significantly upregulated in SD19-vs.-LD19. Additionally, genes encoding cytokinin (CK) dehydrogenase 7 (*CKX7*; Unigene0036599) and cytokinin dehydrogenase (*CYP735A1*; Unigene0029738) were significantly downregulated in SD19-vs.-LD19. *CYTOCHROME P450 734A1* homolog (Unigene0036368), which participates in brassinolide (BR) biosynthesis, was upregulated in SD10-vs.-LD10 and SD19-vs.-LD19; the jasmonate (JA) metabolism-related *JASMONATE O-METHYLTRANSFERASE* homolog (Unigene0020912) and the salicylic acid (SA) metabolism-related *UDP-GLYCOSYLTRAN SFERASE 74F1* homolog (Unigene0004033) were downregulated in SD19-vs.-LD19. A homolog of *CAROTENOID CLEAVAGE DIOXYGENASE 7* (*CCD7*, Unigene0069349) involving in strigolactone (SL) biosynthesis was also identified and showed significant downregulation in SD10-vs.-LD10 (Figure 5C and Supplementary Table S9).

A total of 39 (25.85%) DEGs associated with phytohormone signal transduction of nine hormones were identified and involved 30 phytohormone signal transduction homologs that were associated with signal transduction for nine hormones. Among these DEGs, *GID1B* homologs (Unigene0032780, Unigene0032781, and Unigene0063035), encoding a gibberellin receptor, were upregulated in SD10-vs.-LD10, whereas an *RGL3* homolog (Unigene0071862), encoding a DELLA protein, was significantly downregulated in SD19-vs.-LD19. The ABA signal transduction-related *EID1-LIKE F-BOX PROTEIN 3* (*EDL3*) homolog (Unigene0018152) was upregulated in SD10-vs.-LD10, and *SAUR71* homologs (Unigene0021953 and Unigene0025106), encoding the auxin-responsive protein, were upregulated in SD13-vs.-LD13 and SD19-vs.-LD19. Moreover, in the CK signaling pathway, homologs of *AHPs* (Unigene0034629, Unigene0004315, and Unigene0034630), encoding histidine-containing phosphotransfer protein, were highly expressed in SD10, SD13, and SD19, and an *ARR6* homolog (Unigene0049441), encoding a two-component response regulator, was upregulated in SD19-vs.-LD19. In addition, a *BRI1* homolog (Unigene0024976) in the BR signaling pathway was significantly upregulated in SD7-vs.-LD7; homologs of *MYC4* (Unigene0009399) and *TIFYs* (Unigene0022959 and Unigene0019294) in the JA signaling pathway were upregulated in SD10-vs.-LD10; and a *DWARF14* (*D14*) homolog (Unigene0028658), participating in SL signal transduction, was upregulated in SD7-vs.-LD7 but downregulated in SD19-vs.-LD19 (Figure 5D and Supplementary Table S9).

### Expression Patterns of Genes Associated With Photoperiod Pathways

The photoperiod flowering pathways in plants include the photosensory pathway, the circadian clock, and the systemic effector (Nelson et al., 2009). A total of 10 (6.84%) photoperiod-related homologs were identified. Among these homologs, *CHLOROPHYLL A-B BINDING PROTEIN* (Unigene0075619) was downregulated in SD19-vs.-LD19, whereas *CONSTANS-LIKE 12* (*COL12*, Unigene0039617) and *FD* (Unigene0027311) were upregulated in SD13-vs.-LD13. Meanwhile, homologs of the *FLAVIN-BINDING KELCH REPEAT F-BOX PROTEIN 1* (*FKF1*, Unigene0038380) and the *PRR7* (Unigene0003564) were both downregulated in SD13-vs.-LD13, and a *LUX* homolog (Unigene0011585) was downregulated in SD10-vs.-LD10, whereas homologs of the *nuclear factor Y* (*NF-Ys*; Unigene0025001, Unigene0002375, and Unigene0033157) were upregulated at one or more stages (Figure 5A and Supplementary Table S9).

### Expression Patterns of Genes Associated With the Ambient Temperature Pathway

Plant responses to photoperiod and temperature are coupled (Dong et al., 2020; Meng et al., 2020). The photoperiod-induced floral transition could also affect the expression of a series of ambient temperature-related genes in plants. We identified 28 (19.18%) ambient temperature-related DEGs involving 18 homologs, primarily including the *HEAT STRESS TRANSCRIPTION FACTORS*, *HEAT SHOCK PROTEIN/COGNATE* (*HSPs*), and *pEARLI1*, most of which were highly expressed at several stages under LD (Figure 5B and Supplementary Table S9).

### Expression Patterns of Aging Pathway-Related, Floral Integrator, and Floral Meristem Identity Genes

The aging pathway is an endogenous flowering pathway in plants (Yao et al., 2019). *SQUAMOSA PROMOTER-BINDING-LIKE PROTEIN 4* (*SPL4*) homologs (Unigene0024429 and Unigene0024430) in the aging pathway were upregulated in SD10-vs.-LD10, SD13-vs.-LD13, and SD19-vs.-LD19 (Figure 5F and Supplementary Table S9).

Floral integrators combine environmental and endogenous signals to mediate flowering in plants (Blümel et al., 2015). The floral integrator gene *SOC1* homologs (Unigene0039572 and Unigene0039575) were upregulated in SD10-vs.-LD10, SD13-vs.-LD13, and SD19-vs.-LD19, whereas the *AGL24* homolog (Unigene0049016) was downregulated in SD19-vs.-LD19 (Figure 5G and Supplementary Table S9).

Genetic networks regulating floral transition in plants ultimately activated floral meristem identity genes, thereby causing the transformation from vegetative to floral meristems (Gregis et al., 2006). A total of 15 (10.27%) related DEGs were identified, involving nine floral meristem identity genes (Supplementary Table S9). Among these genes, homologs of *AGL8/FRUITFULL* (*FUL; AGL8*, also known as *FUL*; Unigene0019277, Unigene0004737, Unigene0042052, Unigene0042053, and Unigene0042058), *APETALA 1* (*AP1*; Unigene0019278, Unigene0019279, and Unigene0031106), *LFY* (Unigene0030979 and Unigene0030980), and *SEPALLATAs* (*SEPs*; Unigene0000607, Unigene0034045, and Unigene0025130) were upregulated in one or more developmental stages, whereas homologs of *SHORT VEGETATIVE PHASE* (*SVP*, Unigene0049018) and *TERMINAL FLOWER 1* (*TFL1*, Unigene0026727) were downregulated in SD19-vs.-LD19, SD10-vs.-LD10, and SD13-vs.-LD13 (Figure 5H and Supplementary Table S9).

### Co-expression Network of Floral Transition-Related Genes

A co-expression network constructed using 126 floral transition-related DEGs with edge weights > 0.1 showed 10 hub genes with great connectivity, including homologs of *GLYCERALDEHYDE-3-PHOSPHATE DEHYDROGENASE* (*GAPDH*, Unigene0005846), *AKR1B1* (Unigene0076531), *PKM* (Unigene0073914), *ENOLASE 1* (Unigene0011083 and Unigene0011084), *MED37E* (Unigene0051600), *L-LACTATE DEHYDROGENASE A CHAIN* (Unigene0009368), *HSP83A* (Unigene0031524), *FUL* (Unigene0042052), and *SEP4* (Unigene0025130; Supplementary Figure S8). The genes with the highest network degree were *GAPDH* (Unigene0005846), *AKR1B1* (Unigene0076531), and *PKM* (Unigene0073914), which participated in sucrose and starch catabolism (Supplementary Table S9).

## Discussion

The timing of floral transition in plants is jointly regulated by internal and external environmental cues, of which photoperiod is one of the major environmental factors that affect floral transition in plants (Blümel et al., 2015; Chang et al., 2019). *L. gratissima* is a horticultural ornamental plant with high development potential, and therefore, elucidating the molecular mechanism of its SD photoperiod-induced floral transition is important to its year-round production for commercial purposes. In this study, we conducted transcriptome sequencing of *L. gratissima* shoot apexes and leaves at four stages under LD and SD treatments. A total of 79,870 unigenes were obtained by *de novo* assembly, of which 49.02% were successfully annotated. Currently, there is no report on *L. gratissima* transcriptome assembly and our assembled and annotated transcriptome of *L. gratissima* provides a valuable genetic resource for breeding this species.

### Sugar Signal Mediates Floral Transition in *L. gratissima*

Sugars are an important energy source and participate in floral transition in plants as important signaling molecules (Lebon et al., 2008; Ortiz-Marchena et al., 2015). In co-expression network analysis, all of the first three hub genes (*GAPDH*, *AKR1B1*, and *PKM*) were related to sugar metabolism, implying that sugar might play a vital role in the floral transition process in *L. gratissima*. Leaves are the primary organ of sugar synthesis in plants, and SAMs are the sites of sugar mobilization and consumption, both of which form an important source-sink unit (Bernier and Périlleux, 2005). Floral transition in plants is not only directly associated with sugar content from source and sink but also is regulated by sugar transport (Smeekens et al., 2010). Previous studies have indicated that source-sink regulation could be achieved by the interaction between the bidirectional sugar transporter SWEET and the FT-like protein (Abelenda et al., 2019). In this study, *SWEET16* (Unigene0012661) was significantly upregulated in SD7-vs.-LD7 and SD10-vs.-LD10 (Figure 5E and Supplementary Table S9), indicating that SWEET participated in sucrose transport during floral transition in *L. gratissima*. However, soluble sugars in SAMs decreased from SD0 to SD19 (Figure 3), which is not consistent with the expression profile of genes associated with sucrose metabolism. We speculated that SAM only synthesized limited levels of soluble sugar but *SWEET16* (Unigene0012661) expression in SAMs was only high at SD7 and SD10, and its expression level decreased as SD treatment duration increased (Figure 5E and Supplementary Table S9), subsequently causing a decrease in the rate of the sucrose transport from leaves to SAMs; this suggests that sucrose only acts as an energy source in floral transition in *L. gratissima*.

Trehalose-6-phosphate (T6P) is a component of the plant sugar signaling system and has important effects on flowering and development (Kataya et al., 2020). In *A. thaliana*, the T6P pathway in leaves induced the expression of the florigen gene *FT* in the photoperiodic pathway to affect floral transition, whereas in SAMs, the expression of *SPL* in the aging pathway was controlled by the T6P pathway to directly affect the expression of floral transition-related genes (Wahl et al., 2013). Therefore, the T6P pathway is an important signal that coordinates flowering induction. In this study, except for the T6P synthase homolog *TPS* (Unigene0013555) that was downregulated in SD19-vs.-LD19, other *TPSs* were upregulated at one or more stages during floral transition in *L. gratissima* (Figure 5E and Supplementary Table S9), showing that *TPS* homologs participate in floral transition in *L. gratissima* and the T6P signaling pathway is significantly enhanced during floral transition. *SPL4* was also highly expressed at SD10, demonstrating that T6P in *L. gratissima* SAM promoted floral transition by regulating *SPL4* expression. HK acts as a catalytic enzyme to catalyze hexose phosphorylation, as well as a glucose signal sensor mediating the interaction between the glucose signaling pathway and the ABA signaling pathway to regulate plant development (Moore et al., 2003; Teng et al., 2008). In this study, *HK* homologs (Unigene0044869 and Unigene0044870) were upregulated in SD7-vs.-LD7 and SD13-vs.-LD13 (Figure 5E and Supplementary Table S9). We speculate that HK mainly catalyzed hexose phosphorylation to provide an energy source for initiating floral transition at SD7 and acted as a glucose signal sensor to participate in *L. gratissima* flower development at SD13.

In summary, the sugar metabolism-related genes *TPS* and *HK* entered the flowering regulatory network through the sugar signaling and hormone signaling pathways to regulate floral transition in *L. gratissima*.

### Phytohormones Regulate Floral Transition in *L. gratissima*

Phytohormones play important regulatory roles in plant development and the mechanisms of their participation in floral transition in many plants are extensively studied (Shu et al., 2018; Lin et al., 2019; Zhang et al., 2019; Bao et al., 2020). However, the complex hormone regulatory network of floral transition in perennial woody plants remains unclear. We studied the regulatory patterns of hormones that participate in floral transition in *L. gratissima*.

As one of the most important phytohormones, the function of GA in regulating floral transition is mainly achieved through maintaining GA homeostasis and regulating the levels of DELLA, a growth inhibitor in the GA signaling pathway (Bao et al., 2020). GA homeostasis in plants is maintained through coordinating the expression levels of the GA biosynthesis genes, such as *GA3OXs* and *GA20OXs*, and the catabolic enzyme genes *GA2OXs*, thereby regulating floral transition (Mateos et al., 2015; Bao et al., 2020). In this study, homologs of *GA2OX1* (Unigene0030732) and *GA2OX8* (Unigene0073113) were both upregulated in SD10-vs.-LD10 (Figure 5C and Supplementary Table S9). GA2OXs can catalyze the 2β-hydroxylation of bioactive GAs (such as GA_1_, GA_3_, GA_4_, and GA_9_), resulting in decreased levels of bioactive GAs (Rieu et al., 2008). This may be one of the reasons for low GA_3_ content in shoot apexes and leaves of *L. gratissima*. The main components of GA signaling include the GA receptor GID1B and the growth inhibitors, DELLAs (Bao et al., 2020). When GA concentrations increase, the DELLA protein forms a GA-GID1B-DELLA complex that undergoes degradation by the ubiquitination pathway, thereby regulating the expression of downstream genes (Bao et al., 2020). The GA signaling pathway mainly promotes floral transition by inducing the expression of *SOC1* and *LFY* (Blázquez et al., 1998; Hou et al., 2014; Bao et al., 2020; Fukazawa et al., 2021). In this study, *RGL3* (Unigene0071862) encoding DELLA had low expression in SD10, SD13, and SD19 (Figure 5D and Supplementary Table S9). In contrast, *SOC1* (Unigene0039572 and Unigene0039575) and *LFY* (Unigene0030979) were highly expressed in SD10, SD13, and SD19 (Figures 5G,H and Supplementary Table S9). This showed that low expression levels of the *DELLA* gene *RGL3* could induce the expression of *SOC1* and *LFY*. Additionally, the GA receptor genes *GID1Bs* (Unigene0032780, Unigene0032781, and Unigene0063035) were upregulated in SD10-vs.-LD10 (Figure 5D and Supplementary Table S9), further demonstrating that GA promotes floral transition in *L. gratissima*. However, it may not be GA_1_, GA_3_, GA_4_, or GA_9_ but other active GAs that took effect. Previous studies indicated that GA has a promoting effect in floral transition in *A. thaliana* (Yamaguchi et al., 2014; Bao et al., 2020), whereas GA was found to negatively regulate floral transition in woody plants (Li et al., 2018). GA regulation of floral transition in *L. gratissima* (a woody plant) is similar to herbaceous plants but not woody plants. This unique regulation pattern may be affected by many endogenous and environmental factors, which needs to be further studied in the future.

Other hormones also have some effects in regulating floral transition in *L. gratissima*. ABA is usually considered a stress-related hormone, but it also plays an important role in plant development (Yoshida et al., 2019). However, there is still debate over the role of ABA in floral transition because both promoting and inhibitory effects were reported (Shu et al., 2018; Xiong et al., 2019). In this study, the ABA synthase gene *NCED1* (Unigene0042754 and Unigene0042755) and the catabolic gene *CYP707A1* (Unigene0034382) were both upregulated in SD10-vs.-LD10 (Figure 5C and Supplementary Table S9), and the ABA content in the SAMs was maintained at high levels that initially increased from SD0 to SD13 and subsequently declined, reaching its peak on SD13 (Figure 3). ABF2 is a bZIP transcription factor that binds to ABA. It is also an important component of the glucose signaling pathway (Kim et al., 2004). In this study, *ABF2* (Unigene0046988) was highly expressed in SD10, and likely participated in floral transition in *L. gratissima* by mediating the ABA and glucose signaling pathways. In the ABA core signaling pathway, the protein phosphatase PP2C (ABI1, ABI2, HAB1, and PP2CA/AHG3) acts as a key negative regulatory factor, which has important regulatory effects on the activation of ABA signaling (Tischer et al., 2017). When ABA levels increase in plants, the ABA receptors PYR1/PYLs/RCARs bind and inhibit the phosphatase activity of PP2C, thereby activating the ABA signaling pathway (Tischer et al., 2017). In this study, *PYL4* expression was high in SD13, whereas *PP2C* expression peaked on SD10 but was also high on SD13 (Figure 5D and Supplementary Table S9), suggesting that the activation of the ABA signaling pathway mainly occurred on SD13 and that ABA promoted flower development in *L. gratissima* through the core signaling pathway. EDL3 is a positive regulator of the ABA signal cascade reactions, and it positively regulates the expression of the central component *CONSTANS* (*CO*) in the photoperiod pathway to regulate floral transition (Koops et al., 2011). In this study, the expression of *EDL3* and *COL12* in the photoperiodic pathway peaked on SD10 (Figures 5A,D and Supplementary Table S9), suggesting that ABA promoted floral transition in *L. gratissima* by interacting with EDL3 to induce *COL12* expression.

Plant growth depends on the continuous function of meristems, and CKs have positive effects on SAMs. In this study, the cytokinin synthase gene *LOGs* and the zeatin O-glucosyltransferase gene *ZOG1* were mainly upregulated in SD10-vs.-LD10 and SD13-vs.-LD13 (Figures 5C,D and Supplementary Table S9). It is known that zeatin *O*-glucoside plays important roles in the transport and storage of CKs (Kiran et al., 2012). On the other hand, the *trans*-zeatin synthase gene *CYP735A1* and the cytokinin oxidase/dehydrogenase gene *CKX7* were downregulated in SD19-vs.-LD19 (Figures 5C,D and Supplementary Table S9). Zeatin promotes cell division and has an important role in the early stages of flower bud development and cell division. This is likely the reason zeatin content gradually decreased from SD0 to SD19 (Figure 3). The CK signaling pathway mainly cross talks with AGAMOUS (AG) to regulate SAM differentiation and maintenance (Zhang et al., 2018). RPN12A participates in ATP-dependent ubiquitinated protein degradation, which may inhibit the degradation of one or more factors in CK signaling and balance the proliferation rate of cells during bud development (Ryu et al., 2009). In this study, *AHPs*, which are key components in the cytokinin two-component signaling system (Liu et al., 2017), were highly expressed mainly at SD10, SD13, and SD19; *ARR6*, which is a CK responsive regulator (Liu et al., 2017), was significantly upregulated in SD19-vs.-LD19, and *RPN12A* was upregulated in SD13-vs.-LD13; and moreover, *AGL8* was highly expressed in SD10, SD13, and SD19 (Figures 5D,H and Supplementary Table S9), demonstrating that CK promotes floral transition and flower development in *L. gratissima* indirectly through the effects of AGL8.

In the JA signaling pathway, JAZ (jasmonate-ZIM domain, TIFY family) and MYC2/3/4 regulate floral transition in plants (Bao et al., 2020; Guan et al., 2021). In this study, *TIFYs* and *MYC4* were upregulated in SD10-vs.-LD10 (Figure 5D and Supplementary Table S9), showing that the JA signaling pathway promotes floral transition in *L. gratissima*. In SL signaling pathway, D14 negatively regulates SL signals as an SL receptor (Chevalier et al., 2014). In this study, *D14* (Unigene0028658) expression was high at the early stage of SD treatment, and as treatment duration increased, its expression level decreased (Figure 5D and Supplementary Table S9), which may have been caused by negative feedback regulation of SL signals by D14, thereby regulating SL changes during floral transition in *L. gratissima*. CCD7 is a key enzyme in SL biosynthesis (Bao et al., 2020). Compared with the LD treatment, *CCD7* (Unigene0069349) expression was lower in response to SD treatment and was significantly downregulated in SD10-vs.-LD10 (Figure 5C and Supplementary Table S9), suggesting that SL may inhibit floral transition in *L. gratissima*. In contrast to the results of this study, recent studies have shown that SL inhibits melatonin synthesis, thereby inducing floral transition in *A. thaliana* in an FLC-dependent manner (Zhang et al., 2019). As *L. gratissima* is a perennial woody plant, there may be differences in SL regulatory mechanisms in floral transition compared with *A. thaliana*, which requires further in-depth studies.

YUC-mediated auxin biosynthesis is vital for the formation of floral organs (Cheng et al., 2006). In this study, YUC4 was upregulated in SD19-vs.-LD19 (Figure 5C and Supplementary Table S9), whereas IAA content increased from SD10 to SD13 and continuously decreased afterward (Figure 3), whereas the auxin response gene *SAUR7* was upregulated in SD13-vs.-LD13 and SD19-vs.-LD19. These results suggested that auxin does not participate in regulating floral transition in *L. gratissima* but instead has positive effects on the formation of floral organs.

These hormones interacted with other flowering regulation pathways to further ensure that *L. gratissima* rapidly responded to changes in environmental and endogenous signals to precisely regulate flowering time.

### Flowering Pathways During Floral Transition in *L. gratissima*

The photoperiod pathway is involved in plant response to changes to day length and circadian rhythm, making it one of the most important flowering regulation pathways. In the photoperiod pathways of many plants, the bZIP transcription factor FD forms a transient complex in SAMs with the FT protein from leaves to induce the expression of floral meristem identity genes, thereby promoting floral transition (Abe et al., 2019). In this study, *FD*, *AP1, FUL*, and *AGL8* were highly expressed in SD10 and SD13 (Figures 5A,H), demonstrating that the FD protein directly or indirectly induced the expressions of *AP1, FUL*, and *AGL8*, thereby promoting floral transition in *L. gratissima*. CO is an important regulatory factor in the photoperiod pathway, and the expression of *CO* is regulated by a photoreceptor and circadian rhythm in *A. thaliana*, and when the expression rhythm of *CO* is consistent with the external photoperiod, expression of the downstream gene *SOC1* is activated (Goretti et al., 2020). In this study, *COL12* was upregulated in SD13-vs.-LD13 (Figure 5A and Supplementary Table S9), suggesting that the effects of COL12 in flower development in *L. gratissima* were similar to those of CO in *A. thaliana*.

The transcription factor LUX is one of the components of evening complex (EC) in circadian rhythm and forms the HOS15-EC-HDA9 histone-modifying complex in *A. thaliana* to inhibit *GI* transcription, thereby inhibiting photoperiod-dependent flowering (Park et al., 2019). In this study, *LUX* was downregulated in SD10-vs.-LD10 (Figure 5A and Supplementary Table S9), indicating that LUX had inhibitory effects on floral transition in *L. gratissima*. PRR7 positively regulates *CO* expression to promote floral transition in long-day plants, whereas the *PRR7/PRR3* genes delay floral transition by inhibiting *CO* expression in short-day plants (Nakamichi et al., 2020). In this study, *PRR7* was downregulated in SD13-vs.-LD13 (Figure 5A and Supplementary Table S9), showing that PRR7 inhibits floral transition in *L. gratissima*, which was similar to the other short-day plants. In *A. thaliana*, FKF1 could degrade CDF1 (factor inhibiting *CO* transcription) to regulate *CO* expression and could directly bind to CO, or inhibit *COP1* to stabilize *CO* expression, thereby promoting flowering (Lee et al., 2018). However, *FKF1* was downregulated in SD13-vs.-LD13 (Figure 5A and Supplementary Table S9), which was not consistent with *COL12* expression. This indicated that FKF1 inhibited floral transition in *L. gratissima* and does not interact with COL12, but other mechanisms may be present that require further study. NF-Ys interact with CO in the photoperiod pathway to directly regulate *SOC1* transcription (Hou et al., 2014). In this study, *NF-Ys*, *COL12*, and *SOC1* were highly expressed in SD10 and SD19 (Figures 5A,H), showing that NF-Ys may interact with COL12 in the photoperiod pathway in *L. gratissima* to induce *SOC1* expression, thereby positively regulating floral transition and flowering development in *L. gratissima*.

Previous studies showed that ambient temperature-associated EARLI1 regulated critical genes in the LD photoperiod pathway in *A. thaliana* to promote *FLC* expression and delayed flowering time (Shi et al., 2011). In contrast, *pEARLI1* was upregulated in SD13-vs.-LD13 and SD19-vs.-LD19 in this study (Figure 5B and Supplementary Table S9), indicating that *pEARLI1* promoted floral transition and flower development in *L. gratissima*.

In *A. thaliana*, age signals negatively regulate *miR156* levels to promote *SPL* accumulation (Yao et al., 2019). At SAMs, SPLs target *FUL* and *SOC1* or directly regulate *AP1* transcription to promote flowering (Wang et al., 2009). In this study, *SPL4* was upregulated in SD10-vs.-LD10, SD13-vs.-LD13, and SD19-vs.-LD19 (Figure 5F and Supplementary Table S9), which was consistent with the expression patterns of *SOC1*, *FUL*, and *AP1* (Figures 5G,H), indicating that the aging pathway promoted floral transition and flower development in *L. gratissima* through SPL4-induced expression of *FUL*, *SOC1*, and *AP1*.

The floral integrators SOC1 and AGL24 integrate various flowering signals from photoperiod, temperature, hormone, and age-related signals to activate or inhibit downstream floral meristem identity genes, and ultimately lead to the transformation of vegetative to floral meristems in plants (Blümel et al., 2015). *SOC1* can be indirectly activated by CO (Lee and Lee, 2010). At SAMs, when *SOC1* is activated, SOC1 and AGL24 form a heterodimer to directly activate *LFY* (Lee et al., 2008). In this study, *SOC1*, *AGL24*, and *LFY* were highly expressed in SD10, suggesting that SOC1 and AGL24 can jointly promote *LFY* at this period to promote floral transition in *L. gratissima*. During early flower development, AP1 activates A function to inhibit SOC1 and AGL24 expression to prevent flowering reversion (Lee and Lee, 2010). In SD19, *AGL24* and *SOC1* expression decreased and *AP1* expression increased (Figures 5G,H). These changes may prevent differentiated floral meristems from undergoing flowering reversion.

SEPs are important regulatory factors during flower development and form a heterodimer with AP1 to regulate genes during floral meristem development (Jetha et al., 2014). In this study, *SEPs* were highly expressed in SD10, SD13, and SD9, which was consistent with *AP1* expression (Figure 5H), showing that AP1 mediated positive regulation of floral transition and early flower development in *L. gratissima* by SEPs. In Arabidopsis, SVP is a flowering inhibitor and plays a role in floral transition by directly inhibiting *SOC1* expression at SAMs and leaves (Li et al., 2008). In this study, *SVP* had low expressions in SD10, SD13, and SD19, whereas *SOC1* expression was high (Figures 5G,H), indicating that low levels of SVP induced *SOC1* expression to promote floral transition and flower formation in *L. gratissima*.

TFL1 is a key regulatory factor of floral transition and inflorescence meristem development in *A. thaliana*. TFL1 and FT have highly conserved amino acid sequences but opposite gene functions: FT promotes flowering, whereas TFL1 inhibits flowering (Jin et al., 2020). Previous studies showed that TFL1 negatively regulated transcription of the target gene *FD*, thereby regulating the flowering time and inflorescence meristem development (Hanano and Goto, 2011). In this study, *TFL1* had low expression at SD10 and SD13, which is the opposite of *FD* expression (Figures 5A,H), indicating that low levels of TFL1 promoted *FD* expression and, therefore, floral transition in *L. gratissima*.

**Figure 6:**
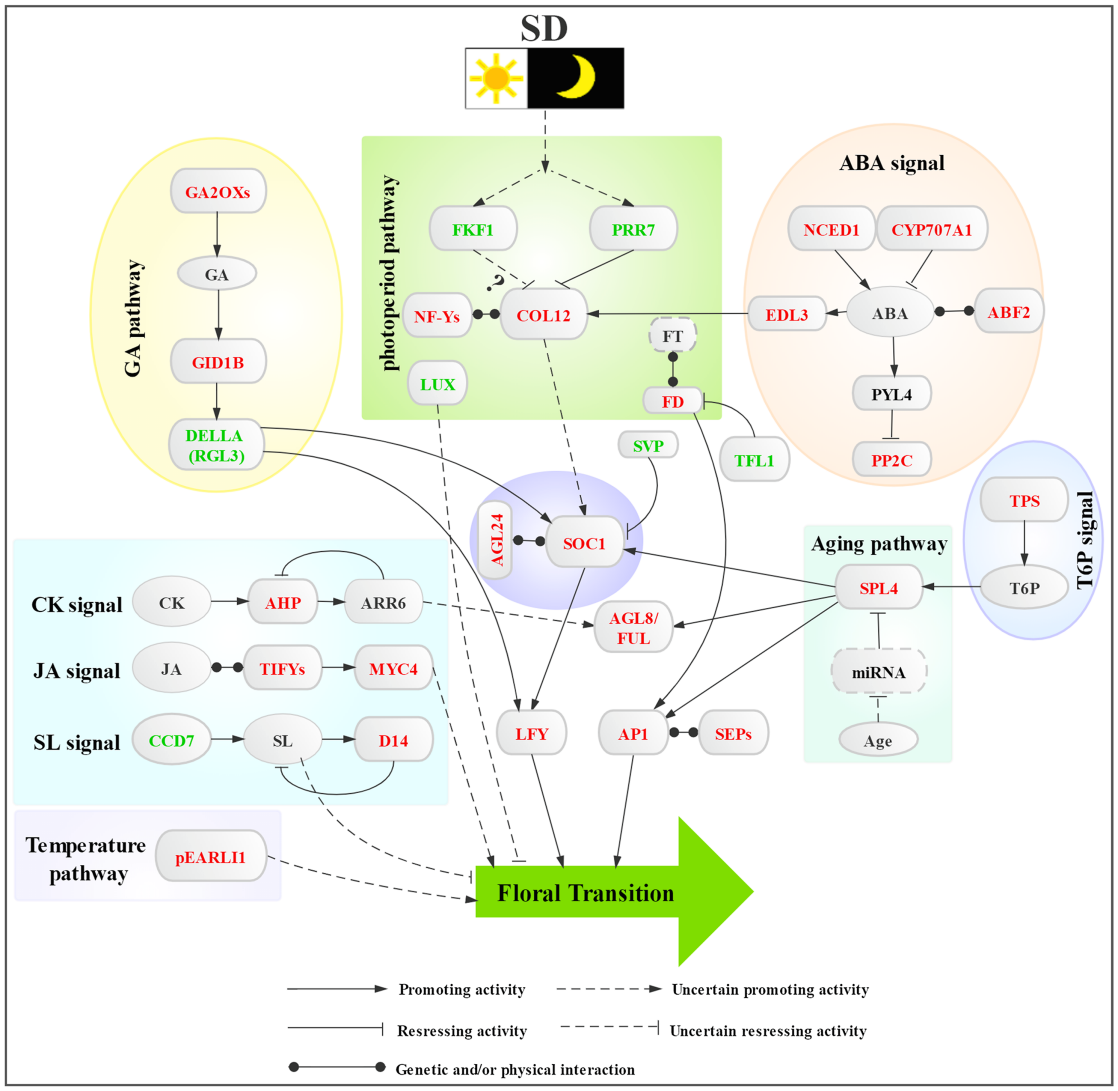
Proposed regulatory network of short-day photoperiod-induced floral transition in *L. gratissima*. Colored fonts represent downregulated (green) or upregulated (red) genes.

Figure 6 shows the hypothetical model of the regulatory network of SD photoperiod-induced floral transition in *L. gratissima*, involved in the regulation of multiple flowering signals in floral transition, including signals for photoperiod, phytohormones (GA, ABA, CK, JA, and SL), sugar, ambient temperature, age, and floral integrator and floral meristem identity genes.

## Conclusion

Our study enables a comprehensive understanding of the gene expression patterns occurring during SD photoperiod-induced floral transition in *L. gratissima*. The histological, endogenous substance contents, and differential gene expression analyzes showed that short-day photoperiod activated systemic responses in *L. gratissima* and induced the generation of flowering signals in the photoperiod pathway. Furthermore, a complex regulatory network, including GA, ABA, CK, JA, and SL signals, sugar signals, and temperature and age signals, was formed through the integration of SOC1 and AGL24. The outcomes of this study will aid in understanding flowering time regulation in *L. gratissima* at the molecular level, provide theoretical guidance for achieving year-round production, and further provide a reference for understanding the regulatory mechanisms of flowering time in other woody plants.

## Data Availability Statement

All datasets generated for this study are included in the article/Supplementary Material. The raw data of RNA sequencing from this study have been deposited into the NCBI Sequence Read Archive (SRA) database (BioProject ID: PRJNA648802). The *de novo* transcriptome has been deposited at GenBank under the accession GIXA00000000.

## Author Contributions

ZL, YW, and HM conceived and designed the study and revised the manuscript. XioL, YW, JA, XZ, YC, and XiuL conducted the experiment and collected the plant materials. XioL and YW analyzed and interpreted the data. XioL and YW wrote the manuscript. All authors read and approved the final version of the manuscript.

## Conflict of Interest

The authors declare that the research was conducted in the absence of any commercial or financial relationships that could be construed as a potential conflict of interest.

## Publisher’s Note

All claims expressed in this article are solely those of the authors and do not necessarily represent those of their affiliated organizations, or those of the publisher, the editors and the reviewers. Any product that may be evaluated in this article, or claim that may be made by its manufacturer, is not guaranteed or endorsed by the publisher.

## Figures and Tables

**Table 1:** 

Term	Definition
ABA	Abscisic acid
AG	AGAMOUS
AGL24	AGAMOUSLIKE24
AP1	APETALA1
BGLU24	BETA-GLUCOSIDASE 24
CAB40	CHLOROPHYLL A-B BINDING PROTEIN
CCD7	CAROTENOID CLEAVAGE DIOXYGENASE 7
CK	Cytokinin
CO	CONSTANS
COL12	CONSTANS-LIKE 12
CYP734A1	CYTOCHROME P450 734A1
D14	DWARF14
DEG	Differentially expressed gene
EC	Evening complex
EDL3	EID1-LIKE F-BOX PROTEIN 3
FKF1	FLAVIN-BINDING KELCH REPEAT F-BOX PROTEIN 1
FT	FLOWERING LOCUS T
FUL	FRUITFULL
GA	Gibberellin
GA2OX1	GIBBERELLIN 2-BETA-DIOXYGENASE 1
GOLS2	GALACTINOL SYNTHASE 2
HK	HEXOKINASE
HPLC-MS	High-performance liquid chromatography-mass spectrometry
HSFs	HEAT STRESS TRANSCRIPTION FACTORS
HSPs	HEAT SHOCK PROTEIN/COGNATE
IAA	Indole-3-acetic acid
JA	Jasmonic acid
JMT	JASMONATE O-METHYLTRANSFERASE
LD	Long day
LFY	LEAFY
NF-Y	Nuclear factor Y
PRR7	PSEUDO-RESPONSE REGULATOR 7
RFS	RAFFINOSE SYNTHASE
SD	Short day
SEP3	SEPALLATA3
SEPs	SEPALLATAs
SL	Strigolactone
SOC1	SUPPRESSOR OF OVEREXPRESSION OF CONSTANS 1
SPL4	SQUAMOSA PROMOTER-BINDING-LIKE PROTEIN 4
SVP	SHORT VEGETATIVE PHASE
T6P	Trehalose-6-phosphate
TFL1	TERMINAL FLOWER 1
TPS	TREHALOSE-6-PHOSPHATE SYNTHSE
UGT74F1	UDP-GLYCOSYLTRANSFERASE 74F1
WGCNA	Weighted gene co-expression network analysis
ZT	Zeatin

## Footnotes

^1^ https://www.plabipd.de/portal/mercator-sequence-annotation
