# Supplementary material for: Morphological, Physiological, and Molecular Responses of Sweetly Fragrant Luculia gratissima During the Floral Transition Stage Induced by Short-Day Photoperiod
Source: Front Plant Sci. 2021 Aug 11;12:715683. doi: 10.3389/fpls.2021.715683 (PMC8385556; doi:10.3389/fpls.2021.715683)
Supplement: Supplementary file 1 [file Data_Sheet_1.ZIP › Supplementary Material and Data/Supplementary Material/Supplementary_Figures_and_Tables_S1-S3.docx]

**SUPPLEMENTARY MATERIAL**

**SUPPLEMENTARY FIGURES**


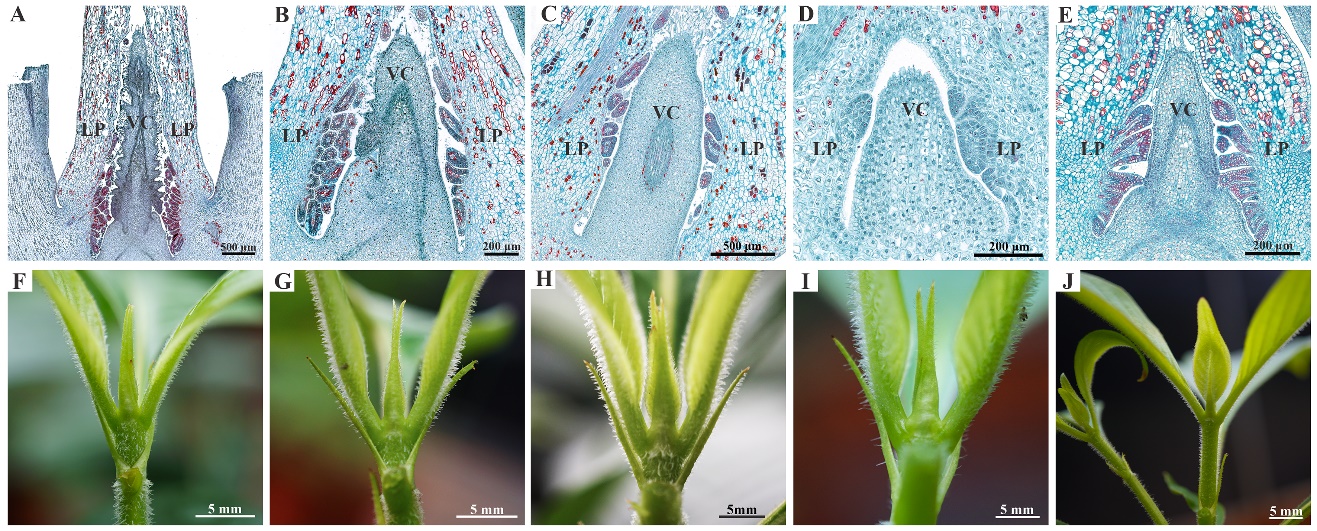


**Supplementary Figure S1 |** *Luculia gratissima* morphological and histological characteristics, shoot apexes at five time points upon long-day treatment. Shoot apexes at 7 d (**A, F**), 10 d **(B, G)**, 13 d **(C, H)**, 19 d **(D, I)**, and 90 d **(E, J)** after the initiation of the LD treatment. (**A, C**) Histological images obtained from paraffin-embedded sectioned samples (scale bar: 500 μm). (**B, D, E**) Histological images obtained from paraffin-embedded sectioned samples (scale bar: 200 μm). (**F-J**) The external morphology of shoot apexes at different developmental stages (scale bar: 5 mm). LP: leaf primordia; VC: vegetative cone.


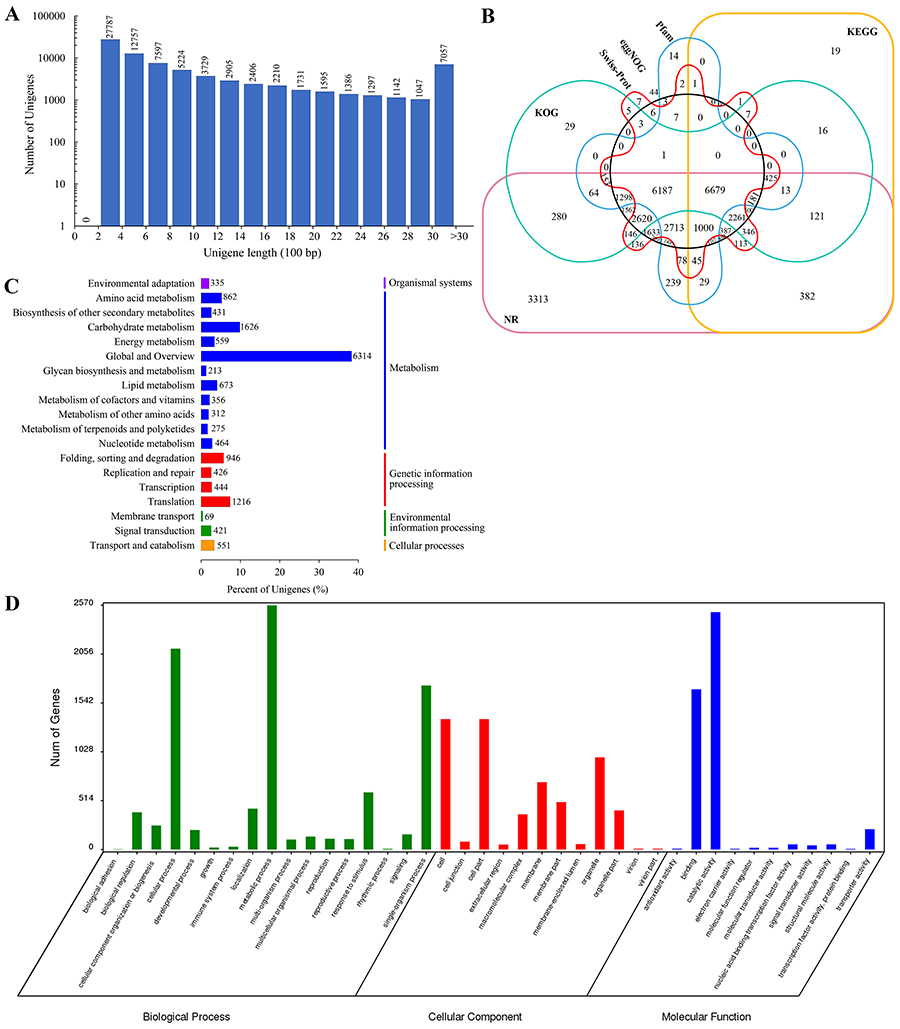


**Supplementary Figure S2 |** Unigene length distribution and annotation statistics. (**A**) Length distribution of the assembled unigenes. (**B**) Venn diagram for the number of unigenes annotated by six different databases – NR, KOG, KEGG, Swiss-Prot, eggNOG and Pfam. (**C**) KEGG classification of unigenes. (**D**) Gene ontology classification of unigenes.


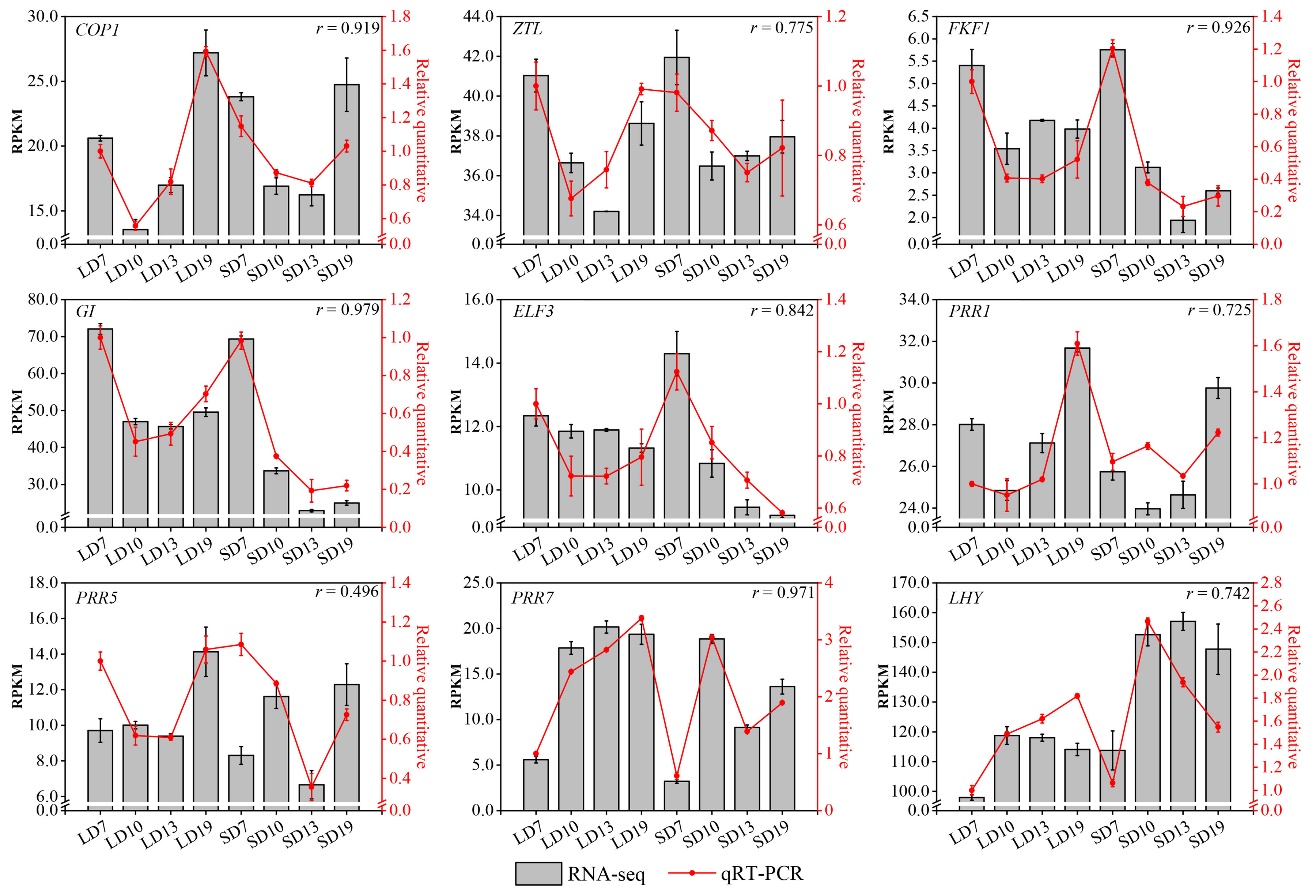


**Supplementary Figure S3 |** Relative expression of flowering-related genes at four time points under short-day and long-day treatments. The relative gene expression was detected by RNA sequencing (RNA-seq) and confirmed by quantitative real-time PCR (qRT-PCR). *r*, Pearson correlation coefficient.


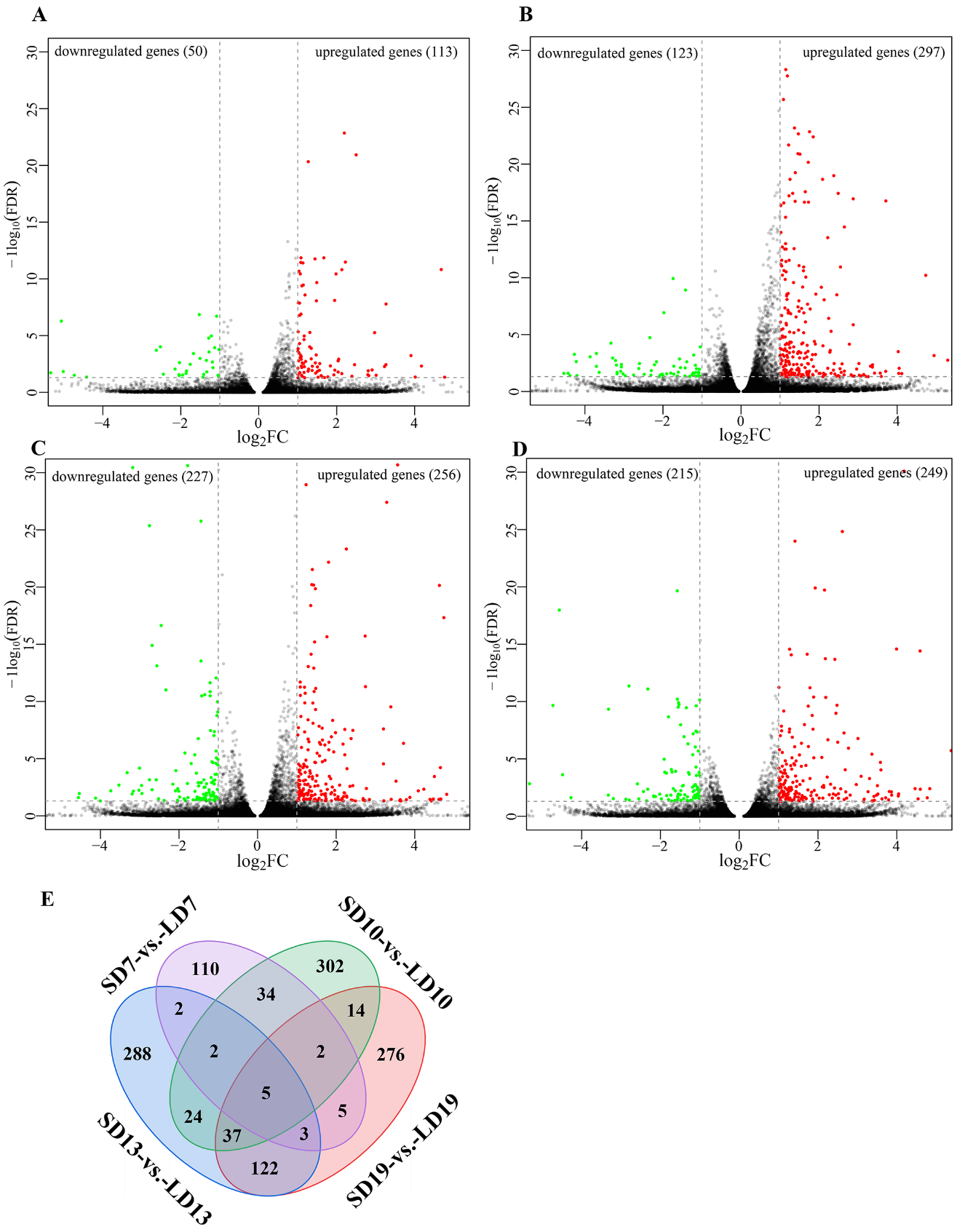


**Supplementary Figure S4 |** Differentially expressed genes (DEGs) in each comparison. Principal component analysis (PCA) of 24 RNA-seq samples in this study. Numbers of DEGs in comparisons (**A**) SD7-vs.-LD7, (**B**) SD10-vs.-LD10, (**C**) SD13-vs.-LD13, and (**D**) SD19-vs.-LD19. (**E**) Venn diagram showing differentially and stage-specific gene profile per comparison.


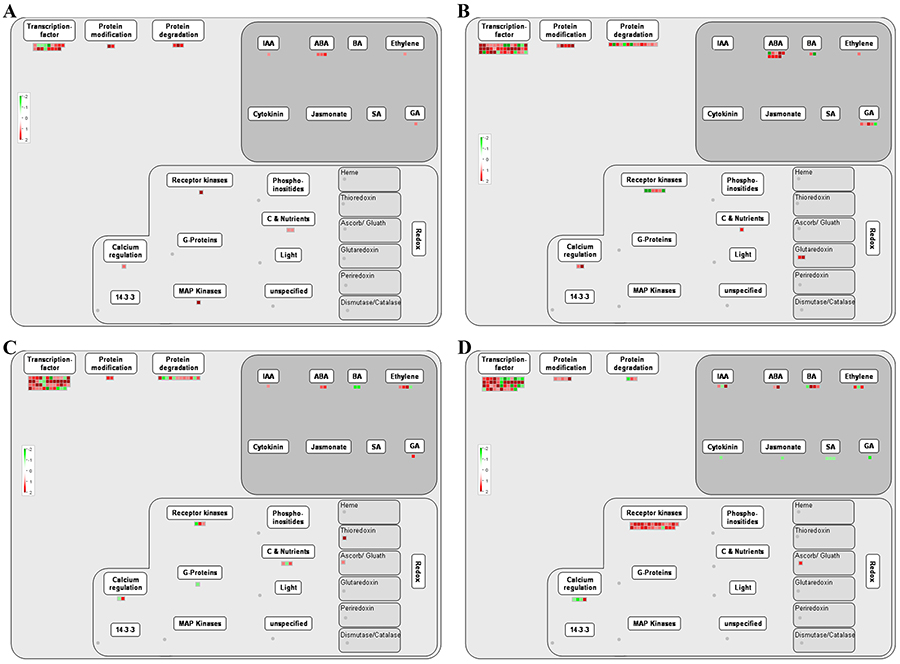


**Supplementary Figure S5 |** Regulation overview in each comparison. The regulation overview map was designed by MapMan based on the transcript levels of differentially expressed genes (DEGs) within (**A**) SD7-vs.-LD7, (**B**) SD10-vs.-LD10, (**C**) SD13-vs.-LD13, and (**D**) SD19-vs.-LD19. The color indicates log_2_fold change, with green and red colors representing down- and up-regulated transcripts, respectively.


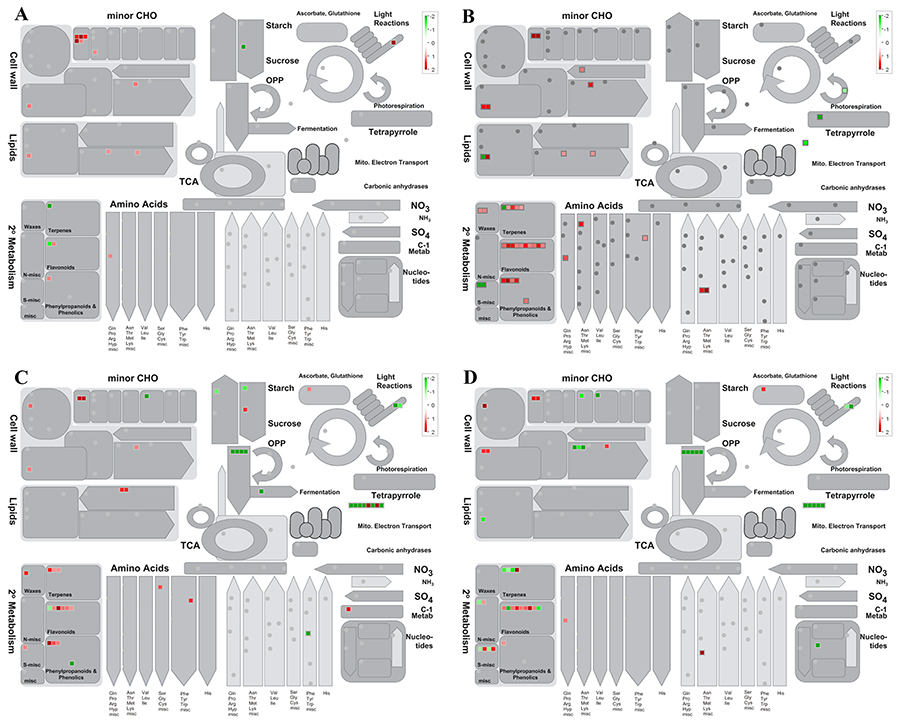


**Supplementary Figure S6 |** Metabolism overview in each comparison. The metabolism overview map was designed by MapMan based on the transcript levels of differentially expressed genes (DEGs) in (**A**) SD7-vs.-LD7, (**B**) SD10-vs.-LD10, (**C**) SD13-vs.-LD13, and (**D**) SD19-vs.-LD19. The color indicates log_2_fold change, with green and red colors representing down- and up-regulated transcripts, respectively.


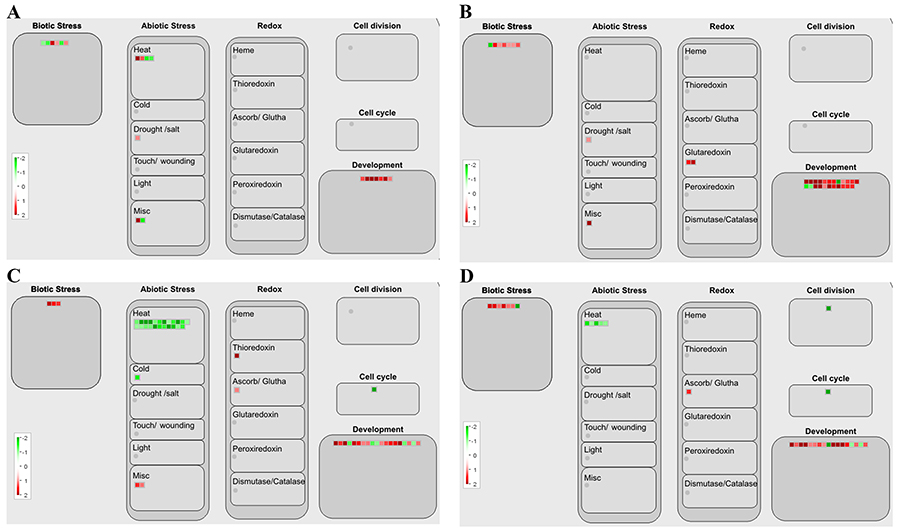


**Supplementary Figure S7 |** Cellular response overview in each comparison. Cellular response overview maps were designed by MapMan based on the transcript levels of differentially expressed genes (DEGs) in (**A**) SD7-vs.-LD7, (**B**) SD10-vs.-LD10, (**C**) SD13-vs.-LD13, and (**D**) SD19-vs.-LD19. The color indicates log_2_fold change, with green and red colors representing down- and up-regulated transcripts, respectively.


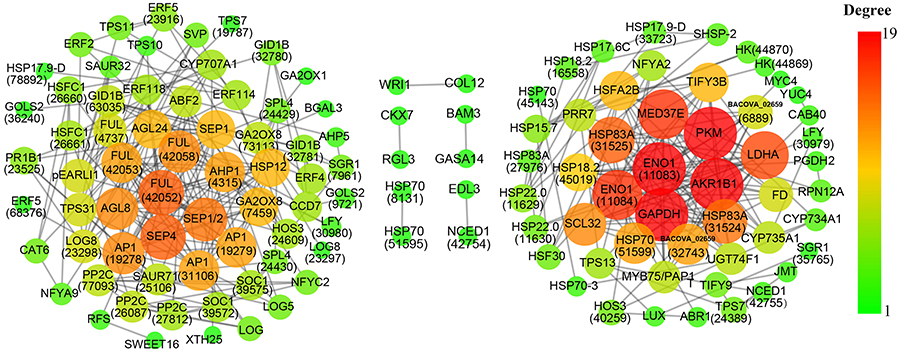


**Supplementary Figure S8 |** Co-expression network of differentially expressed genes (DEGs) associated with floral transition in *Luculia gratissima*. A total of 126 floral transition-related DEGs with edge weight > 0.1 were mapped by Cytoscape. The larger and redder the circles, the higher is the connectivity. When more than two unigenes are the same gene, the unigene ID is marked in parentheses after the gene name.

**SUPPLEMENTARY TABLES**

**SUPPLEMENTARY TABLE** **LEGENDS**

**Supplementary Table S1 |** List of primer sets used in the study

**Supplementary Table S2 |** RNA sequencing (RNA-seq) statistics

**Supplementary Table S3 |** Summary of the assembled unigenes

**Supplementary Table S4 |** Differentially expressed genes (DEGs) at four developmental stages of *Luculia gratissima*. (**a**) DEGs in the comparison SD7-vs.-LD7. (**b**) DEGs in the comparison SD10-vs.-LD10. (**c**) DEGs in the comparison SD13-vs.-LD13. (**d**) DEGs in the comparison SD19-vs.-LD19.

**Supplementary Table S5 |** MapMan annotation of differentially expressed genes (DEGs) at four developmental stages of *Luculia gratissima*. (**a**) MapMan annotation of DEGs for comparison SD7-vs.-LD7. (**b**) MapMan annotation of DEGs for comparison SD10-vs.-LD10. (**c**) MapMan annotation of DEGs for comparison SD13-vs.-LD13. (**d**) MapMan annotation of DEGs for comparison SD19-vs.-LD19.

**Supplementary Table S6 |** Co-expression module analysis for 1,226 differentially expressed genes at four developmental stages of *Luculia gratissima*. (**a**) The kME (module membership-measuring how correlated a gene is to the eigengene), kME-*p*-value, and connectivity (connectivity with all the other genes in the same module) of each gene in the 11 co-expression modules. (**b**) The correlation co-efficient between the module eigengene and the specific stage and *p*-value of the corresponding correlation co-efficient.

**Supplementary Table S7 |** Gene ontology (GO) enrichment analysis of genes specific to each co-expression module identified by weighted gene co-expression network analysis (WGCNA) of *Luculia gratissima*. (**a**) Significantly enriched GO terms of genes specific to each co-expression module. (**b**) Element set of overlapping regions of significantly enriched GO terms in 11 co-expression modules.

**Supplementary Table S8 |** Candidate floral differentially expressed genes (DEGs) uniquely or commonly belonging to each comparison. (**a**) Candidate floral DEGs uniquely belonging to each comparison. (**b**) Candidate floral DEGs commonly belonging to each comparison.

**Supplementary Table S9 |** Significance of candidate floral differentially expressed genes involved in various flowering pathways.

**Supplementary Table S1 |** List of primer sets used in the study

| Gene ID | Gene name | Forward primer (5’-3’) | Reverse primer (5’-3’) | Product size (bp) |
| --- | --- | --- | --- | --- |
| Unigene0031506 | *COP1* | GCAGTAGCAAGAAAGAAGCGTG | GTTCAGCAACAACCCGCAA | 202 |
| Unigene0041339 | *ZTL* | CGGTTGGGAATCGTGTGGTT | AAGCGGAGGGGCTAAACTGG | 286 |
| Unigene0038380 | *FKF1* | CCTGCTGAAGAAAAACCATCCTG | TGCCCACCAAGAACCAATAC | 119 |
| Unigene0051409 | *GI* | CGCAAGATACACAGCAACGG | TGGATGGTGAAGGACGAAAGT | 173 |
| Unigene0051761 | *ELF3* | TGTGCCAAGCCAGAGAAGTG | TGGAAAATGCGAGCGACAGA | 291 |
| Unigene0045946 | *PRR1* | GGCATCATCTCCCTACTACCC | GCTGTTTCCGATTGACATACCTG | 209 |
| Unigene0047475 | *PRR5* | GCCTCCAGACGAGAGTGTT | TCGCTTGACATCGGTTTGC | 234 |
| Unigene0003564 | *PRR7* | GGGAGTGAAAGTGGGATACGG | CACGGTGACATTGGTTGAGG | 209 |
| Unigene0035686 | *LHY* | CACCACAATAACCACGGCAC | TCCTCCTCAGTCCATCGCTC | 228 |
| Unigene0004954 | *ACT7* | GTCCTCTTCCAGCCTTCTATCA | GACCCTCCAATCCAGACACT | 254 |
| Unigene0052171 | *EF1-α* | CGTTGCTTGCCTTTACCCTTG | AATCCAGAGATGGGGACGAA | 178 |

*COP1*: *CONSTITUTIVE PHOTOMORPHOGENIC1*; *ZTL*: *ZEITLUPE*; *FKF1*: *FLAVIN-BINDING KELCH REPEAT F-BOX PROTEIN 1*; *GI*: *GIGANTEA*; *ELF3*: *EARLY FLOWERING 3*; *PRR1*: *PSEUDO-RESPONSE REGULATOR 1*; *PRR7*: *PSEUDO-RESPONSE REGULATOR 7*; *PRR5*: *PSEUDO-RESPONSE REGULATOR 5*; *LHY*: *LATE ELONGATED HYPOCOTYL*; *ACT7*: *ACTIN7*; *EF1-α*: *ELONGATION FACTOR 1-ALPH*

**Supplementary Table S2 |** RNA sequencing (RNA-seq) statistics

| Samples | Raw reads | Clean reads | Clean bases (bp) | Q20 (%) | Q30 (%) | GC (%) |
| --- | --- | --- | --- | --- | --- | --- |
| SD7-1 | 51,881,346 | 51,160,606 | 7,601,480,922 | 99.10 | 97.16 | 43.48 |
| SD7-2 | 58,776,496 | 58,062,056 | 8,628,592,099 | 99.12 | 97.22 | 43.59 |
| SD7-3 | 45,696,612 | 45,063,732 | 6,697,181,825 | 99.10 | 97.16 | 43.49 |
| SD10-1 | 52,569,004 | 51,858,758 | 7,706,661,938 | 99.09 | 97.12 | 43.48 |
| SD10-2 | 49,446,270 | 48,812,602 | 7,251051,347 | 99.10 | 97.17 | 43.42 |
| SD10-3 | 48,748,280 | 48,106,118 | 7,149,047,543 | 99.11 | 97.19 | 43.44 |
| SD13-1 | 47,325,984 | 46,746,098 | 6,952,415,915 | 99.12 | 97.23 | 43.51 |
| SD13-2 | 52,831,042 | 52,131,200 | 7,746,722,199 | 99.08 | 97.11 | 43.51 |
| SD13-3 | 46,347,650 | 45,768,998 | 6,804,500,107 | 99.11 | 97.19 | 43.58 |
| SD19-1 | 57,461,908 | 56,743,110 | 8,435,855,677 | 99.12 | 97.21 | 43.50 |
| SD19-2 | 56,850,250 | 55,978,386 | 8,314,072,935 | 99.01 | 96.93 | 43.47 |
| SD19-3 | 59,712,066 | 58,966,714 | 8,765,419,413 | 99.11 | 97.19 | 43.57 |
| LD7-1 | 41,515,748 | 40,986,320 | 6,091,706,280 | 99.12 | 97.21 | 43.47 |
| LD7-2 | 50,906,436 | 50,271,370 | 7,472,522,142 | 99.13 | 97.23 | 43.54 |
| LD7-3 | 50,254,076 | 49,652,448 | 7,381,356,700 | 99.15 | 97.29 | 43.54 |
| LD10-1 | 64,251,880 | 63,424,520 | 9,424,005,429 | 99.08 | 97.11 | 43.75 |
| LD10-2 | 66,144,496 | 65,287,024 | 9,710,309,242 | 99.13 | 97.23 | 43.54 |
| LD10-3 | 48,782,846 | 48,136,294 | 7,156,525,382 | 99.12 | 97.22 | 43.59 |
| LD13-1 | 47,080,388 | 46,441,192 | 6,903,928,915 | 99.13 | 97.20 | 43.61 |
| LD13-2 | 44,671,830 | 44,072,346 | 6,550,523,204 | 99.15 | 97.27 | 43.62 |
| LD13-3 | 50,963,516 | 50,277,230 | 7,472,701,746 | 99.14 | 97.24 | 43.54 |
| LD19-1 | 46,031,384 | 45,327,894 | 6,733,575,335 | 99.07 | 97.04 | 43.58 |
| LD19-2 | 48,005,318 | 47,349,132 | 7,038,587,587 | 99.15 | 97.25 | 43.51 |
| LD19-3 | 50,171,844 | 49,479,632 | 7,354,964,273 | 99.15 | 97.26 | 43.48 |
| Total or mean | 1,236,426,670 | 1,220,103,780 | 181,343,708,155 | 99.11 | 97.18 | 43.53 |

**Supplementary Table S3 |** Summary of the assembled unigenes

| Total number of unigenes | Total assembled bases (bp) | GC percentage (%) | Mean length of unigenes (bp) | N50 (bp) |
| --- | --- | --- | --- | --- |
| 79,870 | 89,168,958 | 38.02 | 1,116 | 2,118 |
