# Supplementary material for: Morphological, Physiological, and Molecular Responses of Sweetly Fragrant Luculia gratissima During the Floral Transition Stage Induced by Short-Day Photoperiod
Source: Front Plant Sci. 2021 Aug 11;12:715683. doi: 10.3389/fpls.2021.715683 (PMC8385556; doi:10.3389/fpls.2021.715683)
Supplement: Supplementary file 1 [file Data_Sheet_1.ZIP › Supplementary Material and Data/Supplementary_Data.docx]

**SUPPLEMENTARY DATA**

**Evaluation of the optimal reference genes (Unpublished data)**

In order to accurately quantify the expression level of genes, the geNorm software (Mestdagh et al., 2009; Vandesompele et al., 2002) was used to evaluate the four housekeeping genes (*ACT7*, *EF1-α*, *TUB*, *UBQ*) from the transcriptome data of *Luculia gratissima* ‘Xiangfei’. The results showed that the average expression stability values (*M*) from the candidate internal reference genes were *ACT7*/*EF1-α* (0.35) = *ACT7* (0.35) = *EF1-α* (0.35) < *TUB* (0.47) < *UBQ* (0.67), indicating that *ACT7* and *EF1-α*, and their combinations, had the most stable expression. Furthermore, pairwise variation (*V*_2/3_ = 0.144) was lower than 0.15 (Vandesompele et al., 2002), indicating that the optimal number for internal reference genes was two genes. Therefore, the *ACT7/EF1-α* combination was used as internal reference in this study to accurately standardize the expression of candidate flower-related genes.

**REFERENCES**

Mestdagh, P., Van Vlierberghe, P., De Weer, A., Muth, D., Westermann, F., Speleman, F., et al. (2009). A novel and universal method for microRNA RT-qPCR data normalization. *Genome Biol.* 10, R64. doi: 10.1186/gb-2009-10-6-r64

Vandesompele, J., De Preter, K., Pattyn, F., Poppe, B., Van Roy, N., De Paepe, A., et al. (2002). Accurate normalization of real-time quantitative RT-PCR data by geometric averaging of multiple internal control genes. *Genome Biol.* 3, H34. doi: 10.1186/gb-2002-3-7-research0034
